# Recent advances and current challenges of new approach methodologies in developmental and adult neurotoxicity testing

**DOI:** 10.1007/s00204-024-03703-8

**Published:** 2024-03-13

**Authors:** Melania Maria Serafini, Sara Sepehri, Miriam Midali, Marth Stinckens, Marta Biesiekierska, Anna Wolniakowska, Alexandra Gatzios, Elise Rundén-Pran, Edyta Reszka, Marina Marinovich, Tamara Vanhaecke, Joanna Roszak, Barbara Viviani, Tanima SenGupta

**Affiliations:** 1 Department of Pharmacological and Biomolecular Sciences, “Rodolfo Paoletti”, Università degli Studi di Milano, Milan, Italy; 2 Department of In Vitro Toxicology and Dermato-Cosmetology (IVTD), Vrije Universiteit Brussels, Brussels, Belgium; 3 Department of Translational Research, Nofer Institute of Occupational Medicine, Lodz, Poland; 4 The Climate and Environmental Research Institute NILU, Kjeller, Norway; 5 Center of Research on New Approach Methodologies (NAMs) in chemical risk assessment (SAFE-MI), Università degli Studi di Milano, Milan, Italy

**Keywords:** New approach methodologies, Developmental neurotoxicity, Adult neurotoxicity, Adverse outcome pathways

## Abstract

Adult neurotoxicity (ANT) and developmental neurotoxicity (DNT) assessments aim to understand the adverse effects and underlying mechanisms of toxicants on the human nervous system. In recent years, there has been an increasing focus on the so-called new approach methodologies (NAMs). The Organization for Economic Co-operation and Development (OECD), together with European and American regulatory agencies, promote the use of validated alternative test systems, but to date, guidelines for regulatory DNT and ANT assessment rely primarily on classical animal testing. Alternative methods include both non-animal approaches and test systems on non-vertebrates (e.g., nematodes) or non-mammals (e.g., fish). Therefore, this review summarizes the recent advances of NAMs focusing on ANT and DNT and highlights the potential and current critical issues for the full implementation of these methods in the future. The status of the DNT in vitro battery (DNT IVB) is also reviewed as a first step of NAMs for the assessment of neurotoxicity in the regulatory context. Critical issues such as (i) the need for test batteries and method integration (from in silico and in vitro to in vivo alternatives, e.g., zebrafish, *C. elegans*) requiring interdisciplinarity to manage complexity, (ii) interlaboratory transferability, and (iii) the urgent need for method validation are discussed.

## Neurotoxicity testing: why is it challenging?

Until today it has been difficult for toxicologists to completely define what neurotoxicity concretely entails due to the complex structure and function of the nervous system, as well as its intricate interplay with other organ systems (e.g., immune system, endocrine system, and microbiome populating the gastrointestinal tract) (Maurer et al. 2015). The human brain is vulnerable to a wide range of toxic agents, all with their mode of action (MoA) (Sombers and Patisaul 2022). A clear-cut definition describes neurotoxicity as “Any adverse effect on the chemistry, structure, and function of the nervous system during development or at maturity, induced by chemical or physical influence” (Costa 1998). This includes morphological changes like neuronopathy (degeneration of a neuron), axonopathy (axon degeneration), myelinopathy (loss of myelin), and other gliopathies (dysfunctional glial cells, namely microglia and astrocytes), as well as neurochemical changes that lead to impaired function of the nervous system (Giordano and Costa 2012). The complexity of the nervous system makes it difficult to understand the relationship between exposure to environmental factors and the occurrence of neurological dysfunction, which deserves a better understanding of the molecular mechanisms involved (Sombers and Patisaul 2022). Another concept that can be difficult to determine in neurotoxicity is whether an effect on the nervous system is direct or indirect. Secondary effects on the function and structure of the nervous system due to hepatic, renal, pancreatic, or cardiovascular injury, or because of interference with the endocrine system, could also be considered neurotoxic in an indirect way (Costa 1998; Giordano and Costa 2012). Furthermore, a combination of both effects is possible, e.g., a halogenated compound can be neurotoxic in a direct manner as it interacts with the neurons, but also in an indirect manner to the developing nervous system as it alters the thyroid hormone homeostasis (Costa and Giordano 2007; Crofton 2008).

In terms of toxicity, chemical-induced neurotoxicity may be due to a short-term interaction with a target or as a consequence of long-term or repeated exposure. In the former case, the effect may be reversible after cessation of exposure (e.g., solvents) or after reactivation of the target (e.g., organophosphate pesticides, carbamates). In the latter case, the nature, localization (i.e., central or peripheral nervous system), and extent of damage may lead to irreversible effects or favor the progression of complex pathologies, as suggested by epidemiological studies. The overall picture may be further complicated by the possibility that the neurotoxic effect may occur hours or days after exposure (e.g., tri-ortho-cresyl-phosphate) (Spencer and Lein 2024). Acute neurotoxicity that causes severe damage or even death in a laboratory or clinical environment is relatively simple to measure (OECD 1997). It is much more challenging to evaluate the more subtle maladaptive effects of chronic or cumulative exposure, especially during critical windows of neuronal development (OECD 2007). As the developing nervous system could be more susceptible to exposure to hazardous chemicals (Giordano and Costa 2012), DNT may already occur at levels that do not cause acute toxicity. As for ANT, it is important to evaluate long-term exposures not only a single hit but rather multiple subtoxic hits over longer periods of exposure. Epidemiological studies suggest a link between specific environmental factors (e.g., pesticides) and complex neurological diseases of adulthood such as Parkinson’s disease (PD), amyotrophic lateral sclerosis, and Alzheimer’s disease (AD) (EFSA 2014). In addition, due to better diagnosis based on the Statistical Manual of Mental Disorders and the International Classification for Diseases (American Psychiatric Association 2013), environmental factor exposure also seems to have a significant impact on DNT inducing an increased prevalence of neurodevelopmental disorders, including autism spectrum disorder (ASD) and attention deficit disorder (ADD), with or without hyperactivity (ADHD) (Heyer and Meredith 2017). Nevertheless, identifying those environmental causes still poses difficulties without a comprehensive knowledge of the mechanisms involved, or which neuronal systems are most prone to environmental injury (Quaak et al. 2013; Roberts et al. 2019). Furthermore, the inherent limitations of epidemiologic studies do not allow conclusions to be drawn about a causal relationship between exposure to environmental contaminants and the onset and progression of complex diseases, but raise concerns and questions about the adequacy of regulatory studies to inform on complex human health outcomes. In this review, we carefully distinguish between DNT and ANT, and try to provide specific examples for both whenever possible. However, it is necessary to specify that so far there is a big difference between the two in terms of the progress made in the search for NAMs specific to one or the other. The study of DNT is at a more advanced stage, so much so that there will be a specific in-depth study in the chapter on in vitro techniques dedicated to the example of DNT IVB, which groups several assays to support hazard and risk assessment with in vitro models.

Historically, test guidelines issued by the OECD and the US EPA have been the basis for assessing ANT and DNT. For ANT, these test guidelines are based on in vivo methods and focused on clinical observations, functional testing of the sensory and motor system, and neuropathological examination in rodents (OECD 1997; US EPA, 1998). For DNT, both the OECD and the US EPA guidelines are based on perinatal exposures to chemicals in rodents, to assess alterations in neurodevelopmental aspects such as neurophysiological and behavioral parameters (OECD 2007; US EPA 1991; OECD 2018). In particular, the DNT study screens for adverse effects pre- and post-natal on the development and function of the neurological system after exposure in utero and through maternal milk until weaning, and offspring are examined neurologically and behaviorally until adulthood (Makris et al. 2009). It is important to point out that systematic DNT assessment is not a standard requirement within the European Union and the United States, but it is based on a weight-of-evidence approach to determine when testing should be recommended considering only specific triggers (e.g., endocrine disruption concerns, structural similarity to known reproductive toxicants, results from other toxicity studies, and anticipated use and human exposure patterns) (Smirnova et al. 2014). Thus, the amount of chemicals and mixtures tested for DNT is very limited (about 140 compounds in Europe and the US) and a significant deficit in knowledge exists (Fritsche et al. 2017; Crofton and Mundy 2021).

## New approach methodologies: why are alternatives to animal testing needed?

**Fig. 1:**
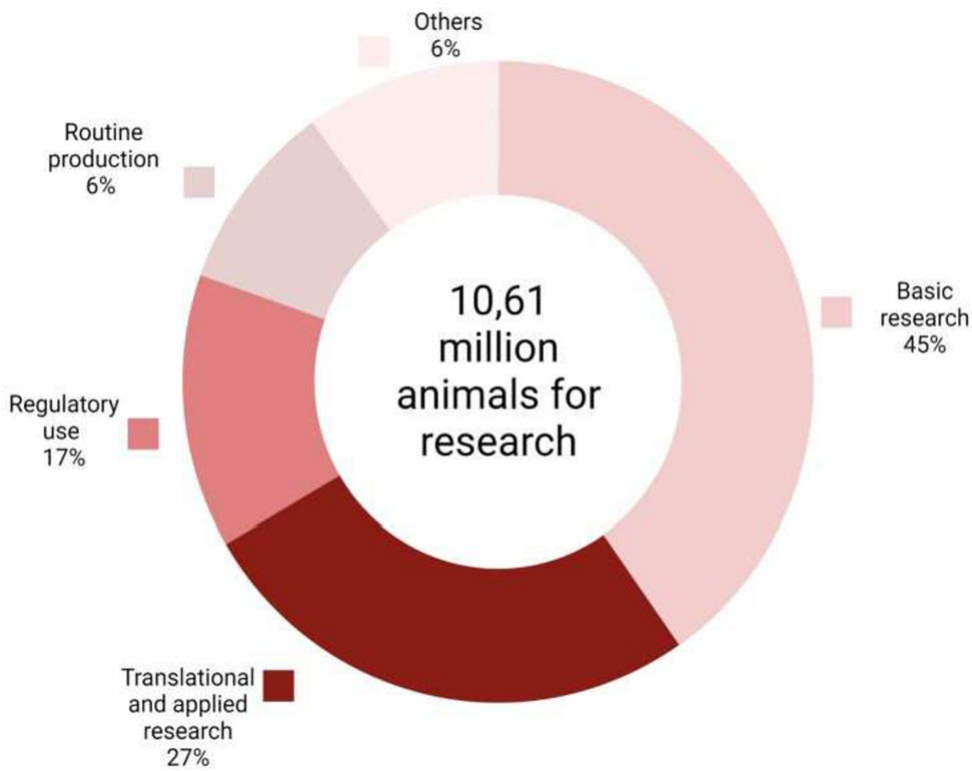
The approximate number of animals used for scientific purposes in the European Union and Norway, including re-uses, in 2019 (data from EURL ECVAM status report 2022 (https://data.europa.eu/doi/10.2760/500414 (accessed on Jan 9, 2023)). Figure created with Biorender.com

Animal research has undoubtedly contributed to a better understanding of various physiological and pathological conditions. Although there is a visible decrease in the number of procedures on live animals, data from 2011^1^ and 2019^2^ showed that over 10 million animals are still used in scientific research (Fig. 1) with rodents being the most used species and representing, together with rabbits, 80% of the total number of animals used. In recent years, ethical concerns about animal testing have risen regarding the possible advancement of scientific knowledge, animal protection, and especially the relevance of data obtained in animal models to human health for regulatory purposes. Many literature data show a lack of correlation between animal models and humans, as suggested by drugs ineffective in patients although successful preclinical studies (Atkins et al. 2020; Ransohoff 2018). The same is true for toxicants and involves several sources of uncertainty affecting extrapolation to humans.^3^

The 3Rs introduced by Russel and Burch (Russell and Burch 1959) denote Replacement, Reduction, and Refinement, and have had a significant impact on the practice of scientific research.

In terms of replacement (i.e., the usage of methods enabling the absolute replacement of animals), most of the in vitro models require less time and resources for chemical toxicity screening, are more cost-effective, environmentally friendly, avoid species-specific issues (Balls 2002), and in some cases provide more relevant information compared to whole animals (Barbosa et al. 2015). For instance, in vitro cell culture is a good model for early-stage compound prioritization. Using a human cell-based DNT IVB, Klose and colleagues extrapolated benchmark concentrations of flame retardants human exposure via breast milk and suggested low risk for individual compounds. This could raise a potential concern for real-life mixture exposure, especially when different compounds converge through diverse MoA on common endpoints (Klose et al. 2022). These are examples of full replacement (often collectively referred to as non-animal methods), while we refer to partial replacement when we use animals that are not considered to be capable of suffering based on current scientific knowledge (e.g., nematodes, fish) or primary tissues and cells explanted from animals killed solely for this purpose.

Concerning reduction (i.e., minimizing the number of animal individuals while maximizing the information obtained), careful selection of study design and proper application of statistical information allows minimization of animals used while still providing meaningful scientific results with robust and reproducible findings. Methods that maximize the information obtained from each animal are some imaging techniques (e.g., magnetic resonance imaging, positron emission tomography, computer tomography), blood micro-sampling, and omics technologies (Hartung and McBride 2011). Indeed, one recent study found that in response to neurotoxic compounds (i.e., acrylamide, chlorpyrifos, fluoxetine, methyl mercury, and valproic acid), RNA sequencing data predicted changes in the neuronal differentiation pathway related to neural progenitor proliferation, neuronal and glial differentiation, axon development, synaptogenesis, synaptic transmission, and apoptosis in human neuronal progenitor cells (de Leeuw et al. 2022). Thus, while traditional toxicology tests generate knowledge about apical adverse outcomes in experimental animals, omics technologies provide evidence about why an adverse outcome is likely to occur, enabling systems toxicology (Hartung et al. 2017). Omics technologies increase predictive power when incorporated into animal study designs, but sharing data and resources between different research groups is a key issue in this kind of application.

The refinement^4^ (i.e., minimizing pain, suffering, or potential long-lasting harm to the animals) mainly refers to the modification of breeding and experimental procedures to minimize or eliminate pain and distress as well as to improve the welfare of animals. Performing more breeding procedures simultaneously may be beneficial in terms of decreasing the handling stress in some species. In primates, positive reinforcements and operant conditioning are other ways to reduce the potential distress.^5^ Improving the lives of animals is important for the reliability of scientific results, and to break the vicious cycle in which less reliable outcomes (due to stress) lead to unreproducible results and to an increase in the number of animals used (Hendriksen 2009).

Nowadays, neurotoxicity research is shifting from predominantly animal testing to the use of NAMs (Zavala et al. 2020). However, the umbrella term “NAMs” remains raw-edged, as there is no globally standardized definition of this word. Several agencies like the European Chemical Agency (ECHA), the US EPA, the Interagency Coordinating Committee on the Validation of Alternative Methods (ICCVAM), and the Canadian Chemicals Management Plan (CMP) all describe this coined term in their own words (Table 1). Therefore, clarifying and standardizing the definition of the term may be key to defining its use for further research in the field of harmonization.

**Table 1:** The definition of NAMs according to ECHA, US EPA, ICCVAM, and the CMP

ECHA^a^	“NAMs denote alternatives to traditional toxicity methods that typically involve animal testing. These alternatives are useful for predicting and assessing chemical risks and hazards, by providing mechanistic information for biologically complex endpoints. They include, e.g., in vitro, in chemico methods and in silico computational models, which may be used alone or in combination with other methods and have the potential to be quicker, cheaper and use fewer animals”
US EPA^b^	“NAM is broadly descriptive reference to any non-vertebrate animal technology, methodology, approach, or combination thereof that can be used to provide information on chemical hazard and risk assessment. NAMs are functionally equivalent to alternatives to mammal testing”
ICCVAM^c^	“NAM has been adopted as a broadly descriptive reference to any alternative test method or methodology that can be used to provide information on chemical hazard and risk assessment. These new approaches include IATAs, defined approaches for data interpretation and performance-based evaluation of test methods. In this context, alternative test methods include non-animal test systems and phylogenetically lower species, methods that reduce the number of animals required for a specific test or refine animal use to lessen or avoid pain and distress”
CMP^d^	“NAMs are broadly defined as any technology, methodology, approach or combination thereof that can be used to replace, reduce or refine animal toxicity testing and allow for more rapid or effective prioritization and/or assessment of chemicals. These methods may include the use of computer-based (computational) models, modernized whole-organism assays or assays with biological molecules, cells, tissues or organs, as well as exposure prediction approaches. While some NAMs may still make use of animals (zebrafish embryo or 5-day rodent transcriptomics study), the methods are refined to provide new mechanistic knowledge and associated dose–response data. This is an important step toward reducing the total number of animals used in research, product development and chemical risk assessment until the necessary NAMs become available to replace animal toxicity testing”

^a^https://echa.europa.eu/documents/10162/21184118/2023_06_01_nam_workshop_background_note_en.pdf/18873078-7ef6-80d3-b929-3b5a782949c9?t=1684296040053 (accessed on Oct 4, 2023)

^b^https://www.epa.gov/chemical-research/epa-new-approach-methods-efforts-reduce-use-vertebrate-animals-chemical-testing (accessed on Sep 12, 2023)

^c^https://www.oecd.org/chemicalsafety/risk-assessment/concepts-and-available-guidance-related-to-integrated-approaches-to-testing-and-assessment.pdf (accessed on Oct 4, 2023)

^d^https://www.canada.ca/en/health-canada/services/chemical-substances/fact-sheets/use-new-approach-methods-risk-assessment.html (accessed on Sep 12, 2023)

*IATA* Integrated Approaches to Testing and Assessment

## NAMs and neurotoxicity assessment

The human relevance of the results obtained with the complex in vivo regulatory animal studies required by OECD and US EPA guidelines is questionable since significant differences exist between rodents and humans which hampers the extrapolation of the results, as discussed above (Tsuji and Crofton 2012). For this reason, many efforts are being made to develop NAMs that are applicable to the study of DNT and ANT. The data obtained from NAMs are collected in IATA to support chemical safety assessment. The database^6^ on alternative methods to animal experimentation (DB-ALM) of the European Union Reference Laboratory for alternatives to animal testing (EURL-ECVAM) collects the summaries and protocols of methods submitted for validation. If we search for “Neurotoxicity”, the database retrieves 16 entries from a total of 370 entries. In addition, the European Union’s Joint Research Center (JRC) website contains a constantly updated list^7^ of validated methods, but as you can see by scrolling through the topics, no test has yet been approved for neurotoxicity assessment.

Here we review the recent advances of NAMs for ANT and DNT assessment considering computational tools, in vitro cell-based models, *C. elegans*, and zebrafish, intending to highlight the potential and current critical issues for the full implementation of these methods in the future. We also review the current status of NAMs in the regulatory context with a focus on the DNT IVB, which could provide useful data for hazard characterization and risk assessment.

### Adverse outcome pathways (AOP)

An AOP is a conceptual framework in individual or network settings to identify changes sufficient to serve as the basis of hazard assessment (i.e., molecular initiating events, key events, and adverse outcomes), data gaps and to establish testing strategies for regulatory endpoints. Details of AOP components have been extensively reviewed elsewhere (Hemmerich and Ecker 2020; Schultz and Watanabe 2018; Bal-Price et al 2015; Sachana et al 2021b) and will not be discussed in detail here. In toxicology, AOPs/AOP networks are designed to facilitate the adoption of a mechanistic approach in regulatory and epidemiological studies that, as described in Chapter 1, raise the question of a possible link between exposure to chemicals (e.g., pesticides) and the occurrence of complex nervous system pathologies that are difficult to study in animal models. The core of AOPs development is data collection and analysis to define the confidence in the relationship between KERs based on both the biological plausibility and empirical support of KERs (i.e., dose, temporal, and incidence concordance), the confidence and precision of KE measurement, and any identified inconsistencies, uncertainties, and data gaps. All these considerations must be captured in the overall weight of evidence of an AOP that delineates the confidence in the AOP to support regulatory application. As such, AOPs and their network should facilitate the functional understanding of complex pathways and provide a mechanistic basis for the development of an IATA that combines assays and/or predictive models that address events sufficient to measure the hazard of a chemical. There are currently 43 AOPs on the AOP wiki that address neurotoxicity. Many of these lack detailed descriptions or contain minimal information. While it is possible to pinpoint AOPs related to developmental or adult neurotoxicity, there is often insufficient information on the temporal progression of effects and the specific brain regions affected. This dearth of data makes it challenging to identify the precise type of toxicity within the extensive range of functional responses to chemical perturbation outlined in Chapter 1. An exception is represented by the OECD-endorsed AOPs (for ANT AOPs: 3, 10, 48; for DNT: 12, 13, 17, 42, and 54). A notable example is the AOP entitled “Inhibition of mitochondrial complex I of nigro-striatal neurons leads to Parkinsonian motor deficits” (ID 3 in the AOP wiki, the repository of AOPs coordinated by the OECD), which considers the long-term evolution of toxicity (overall table in AOP wiki) together with the vulnerable area/neurons relevant to the development of the targeted pathology. This AOP provides mechanistic plausibility and aids in establishing a causal connection in support of epidemiological observations linking pesticide exposure to an increased risk of developing Parkinson’s disease (Ockleford et al. 2017), an endpoint not routinely captured in regulatory studies. This AOP has been a starting point to inform testing strategies for hazard assessment of different pesticides (Tebby et al. 2022; van der Stel et al. 2020; van der Stel et al. 2021) and to define the IATA case study of the OECD IATA program.^8^ So far, the AOP-based approach has proved useful in integrating specific technologies and test systems which, once coupled with toxicokinetic simulations, have allowed to support read-across of structurally related substances (van der Stel et al. 2021) and to provide an in vitro point of departure for a potential risk of parkinsonian motor deficits after long-term exposure to tebufenpyrad (Alimohammadi et al. 2023). Although these results are very promising in the context of reducing the use of animals and overcoming the limitations of regulatory studies to inform human health outcomes, it is recommended that further case studies be conducted to delineate the applicability and to facilitate and validate a set of best practices (Alimohammadi et al. 2023). The key element in the use of AOP 3 is the high quality, based on the richness of the documentation and the overall assessment of the evidence, which allowed the OECD endorsement.

Some of the most mature AOPs were organized into a network (AOPN) and analyzed for topological features to identify the most connected and studied KEs (nodes) and to provide an overview of the pathways leading to AOPs relevant for ANT and DNT (Spinu et al. 2019). The organization of linear AOs into networks and their analysis aims to provide guidance on which tests to prioritize, support the identification of biomarkers, and may provide an approach to modeling quantitative AOs (Spinu et al. 2019). The benefits, limitations, and challenges of AOPN with a focus on neurotoxicity are discussed in detail by Spinu et al. (2019).

### Computational toxicology

Over the years, with advancements in computational modeling, different techniques and the integration of different types of data have been used and tested to predict ANT and DNT. Computational toxicologists analyze chemical structures to identify toxicity-associated patterns, using structural alerts as warning signs for potential hazardous properties. A structural alert in a chemical compound suggests the presence of certain characteristics or specific metabolic reactions that may lead to toxicity. Different series of structural alerts as well as QSAR models have been developed for ANT (Cronin 1996; Estrada et al. 2001; Grigorev et al. 2018; El Yazal et al. 2001). Moreover, neurodegenerative diseases such as AD, have been studied using human-based high-throughput imaging computational-based analysis to decipher the epigenetic and molecular mechanisms driving disease development. The absorption, distribution, metabolism, and excretion of chemicals involved in either AD development, such as chlorpyrifos linked to Aβ deposition, or compounds for treating AD can be predicted using computational models employed in toxicology. These models, such as in vitro–in vivo extrapolation and physiologically based pharmacokinetic and pharmacodynamics modeling, allow us to define the kinetics and dynamics of compound exposure and forecast long-term chemical effects (Pistollato et al. 2015). In studies regarding DNT, computational models such as probabilistic modeling are being implemented to formulate mechanistically driven hypotheses on how exposure to various environmental chemicals affects the human exposome (Pistollato et al. 2020; Mian et al. 2021). The Bayesian hierarchical model predicts the potential to promote DNT with an accuracy of 76%, classifying the compounds into three probability classes: low, medium, and high. This classification and the methodology are explained further (Spinu et al. 2022). Below, is a description given of the approaches used in computational toxicology in NAMs development for neurotoxicity assessment.

#### In silico tools

Non-testing approaches, commonly referred to as in silico tools, play a crucial role in the development of NAMs. This broad range of applications encompasses the creation and organization of data libraries for efficient data retrieval, as well as the identification of chemical activities (Crofton et al. 2022). In terms of structure–activity identification, three different leading technologies stand out: category formation (grouping) for read-across, (quantitative) structure–activity relationship (Q)SAR (Cronin et al. 2017), and physiologically based kinetic (PBK) models. These technologies offer diverse parameters linking a chemical’s biological activity to its structure based on the similarity principle. Briefly, any molecular descriptor or a set of descriptors can be used to extrapolate information about less-known chemicals, providing insights into their chemical characteristics (Kasteel and Westerink 2021). It should be mentioned that abiotic molecular docking, an example of an *in chemico* testing approach, enhances our understanding of a chemical’s intrinsic ability to interact with macromolecules. The outcomes of *in chemico* testing contribute to the organic chemistry knowledge underlying these interactions. Considering toxicity, the origin of the effect is often the covalent binding to macromolecules. Therefore, an *in chemico* approach offers insights into the intrinsic reactivity of chemicals. These findings are fed into in silico methods, generating computational tools used for screening purposes (Cronin et al. 2009).

In the assessment of adverse neurotoxicity (ANT) and developmental neurotoxicity (DNT), in silico approaches have been applied across various levels of risk assessment, spanning hazard assessment, mechanistic profiling, and predictions of absorption, distribution, metabolism, and excretion (ADME), with a specific emphasis on blood–brain barrier (BBB) models (Jiang et al. 2020; Worth et al. 2011; Wijeyesakere et al. 2020; Chushak et al. 2018; Han et al. 2019). Despite these advancements, the complexity of ANT and DNT as endpoints introduces uncertainties regarding the underlying mechanisms. This complexity is particularly pronounced in the case of DNT, given its time-sensitive nature, where the exposure window further complicates the understanding of toxicity mechanisms. Nevertheless, governmental and non-governmental organizations have compiled data libraries and accessible resources to facilitate the development of NAMs for ANT and DNT. Examples include the Alternative Assessment Dashboard Hazard Database^9^ encompassing over 290,000 hazard data records, the SIDER database, containing data on 1430 drugs and 5880 adverse drug reactions (ADRs), as well as ToxCast and ToxRefDB. It is important to note that while these libraries provide valuable data, none are exclusively designed for ANT and DNT, encompassing various types of toxicity, including endocrine disruption, reproductive toxicity, and chronic organ-specific systemic toxicity (Kuhn et al. 2016). Table 2 summarizes some of the publicly available resources from the National Toxicology Program (NTP) of the United States Department of Health and Human Services.^10^

**Table 2:** A non-exhaustive list of data and resources offered by the National Toxicological Program to researchers and the general public with a short description

Type of resource	Name	Description
Study data	Chemical effects in biological systems (CEBS)	public data repository of toxicogenomics data, clinical data, histopathology findings, microarray and proteomics data over 2000 NTP studies permitting data integration and cross-study analysis and data query using study condition and the subject responses
	NTP report series	Rodent toxicology studies results reviewed by NTP internally. The pathology results are peer-reviewed by the Pathology Working Group (PWG). The data include toxicology (TOX) reports of short-term or sub-chronic studies, technical reports (TR) of long-term or chronic studies and results using genetically modified models (GMM), developmental and reproductive toxicology (DART) studies, immunotoxicology (IMM) studies and peer-reviewed manuscripts published in scientific journals
	NTP historical controls database	A historical summary collection of chronic animal studies and genetically modified models and DART data
Tools and resources	Developmental NeuroToxicity data integration and visualization enabling resource (DNT-DIVER)	A web-based tool to analyze, compare, and visualize multiple DNT assays
	Integrated chemical environment (ICE)	Curated TOX21 data with in silico prediction of physicochemical and ADME properties, mapped in vitro data, and IVIVE tools estimating in vivo exposure (Bell et al. 2020)
	Test method development resources	Recommended protocols, tools, and research opportunities as well as guidelines and regulatory needs (only US), supporting the development of alternative test methods
	TOX21 toolbox	It contains tools for accessing, visualizing, and analyzing quantitative high-throughput screening (qHTS) data library of 10 K. The data library, one of the largest libraries constructed ever, generated 100 million data points from ∼8500 chemicals in more than 70 high-throughput toxicity assays (Richard et al. 2021)
Pathology resources	Non-neoplastic Lesion Atlas	High-quality images of rodents’ body systems and description after exposure to environmental chemicals inducing non-cancer diseases (Schmidt 2014)
	NTP archives	Provides primary public resources for toxicological research including study collections, educational material, and training material on rodent pathology. It contains millions of histological slides and paraffin-embedded tissue blocks; 242,000 bags of formalin-preserved tissues, 74,000 frozen specimens and millions of paper data and microfiche with over 18,000 digital histopathology images

*NTP* National Toxicological Program, *PWG* Pathology Working Group, *TOX* toxicology, *TR* technical reports, *GMM* genetically modified models, *DART* developmental and reproductive toxicology, *IMM* immunotoxicology, *ADME* absorption, distribution, metabolism, excretion, *IVIVE* in vitro to in vivo extrapolation, *qHTS* quantitative high-throughput screening, *CEBS* Chemical Effects in Biological Systems, *DNT-DIVER* Developmental Neurotoxicity Data Integration and Visualization Enabling Resource, *ICE* Chemical Integrated Environment

Except for DNT-DIVER, the resources listed in Table 2 are not specifically tailored to in silico approaches for ANT and DNT but rather encompass other toxicological endpoints and alternative methodologies. These include targeted in vitro testing approaches and the development of in vitro to in vivo extrapolation (IVIVE) tools. In addition, ECHA provides a dossier of all registered substances in Europe, including neurotoxicants. While this data serves as a valuable starting point for retrieving compound-specific existing data, the database lacks interactivity, and access to dossiers is limited to individual retrieval. Another noteworthy database is DevTOX, designed to enhance and standardize the assessment of developmental findings and categorization, with a recent emphasis on developing prediction systems for DNT (Marx-Stoelting et al. 2021).

#### Read-across and category formation

Developing databases for screening and prioritizing chemicals for ANT and DNT based on evidence from both human and animal studies, as well as similarities in functional groups and constituents (chemistry) and biological activity is instrumental in filling data gaps and forming categories. In recent years, significant efforts have been devoted to identifying uncertainties and enhancing the transparency of read-across assessment. Various bodies have developed guidelines and frameworks to facilitate this process (Schultz and Cronin 2017; OECD 2014; ECHA 2008; ECETOC 2014). The use of analogs and read-across concepts to bridge knowledge gaps has been extensively discussed and studied, particularly for neurotoxicants with a known MoA such as the inhibition of complex I or III of the mitochondrial respiratory chain and chemical causing PD-associated neurological effects (van der Stel et al. 2021; OECD 2021; Soares et al. 2022). In 2018, the EPA launched the GenRA (generalized read-across) tool, integrated into the EPA Computational Toxicology Dashboard. This tool predicts analogs based on chemistry and/or bioactivity descriptors using the chemical biological read-across (CBRA) approach (Helman et al. 2019; Hemmerich and Ecker 2020). User-friendly tools like these can be explored for hazard identification and guiding risk minimization strategies with known neurotoxicants.

#### (Quantitative) Structure–activity–relationship (Q)SAR

Machine learning techniques on large data have facilitated the establishment of several QSAR models for neurotoxicants, utilizing various molecular descriptor packages and machine learning algorithms. The construction, validation, and evaluation of these models have been extensively assessed and reported in the literature (Jiang et al. 2020; Worth et al. 2011; Cronin 1996; Nicolotti et al. 2014; Estrada et al. 2001; Malygin et al. 2003). Software-based tools are available for predicting ANT and DNT (e.g., Derek Nexus, PALLAS HazardExpert, and PASS). A better understanding of the mechanisms underlying the toxic effects of chemicals involves identifying the structural fragments responsible, known as structural alerts. Mechanistically based, a group of structural alerts can be used to develop in silico profilers for the early screening of chemicals. Profilers, grouped based on a shared mechanism of toxicity, can flag chemicals linked to specific toxicities in large databases. Nelms et al. developed an in silico profiler for mitochondrial toxicity based on scientific knowledge of mitochondrial structural alerts (Nelms et al. 2015). Mitochondrial respiratory chain dysfunction, studied in vitro and in vivo, has been suggested to trigger various neurotoxicities, including Parkinsonian motor deficits (Delp et al. 2021; Li et al. 2019; Capela and Carvalho 2022).

Furthermore, the web-based expert system, SApredictor^11^ developed by Hua and coworkers, assesses various toxicological endpoints, including neurotoxicity. Using data for 22 toxicity endpoints, they focused on neurotoxicity using Jiang et al. 2020’s collection of data on 495 compounds in humans from ChemIDplus (Jiang et al. 2020). Employing frequency analysis SARpy and fingerprints filter, they identified 18 structural alerts for neurotoxicity in the training dataset. A neurotoxicity computational model based on 684 annotations (329 positives and 355 negatives) was established with the applicability domain calculated using the Tanimoto coefficient in the similarity matrix based on the Klekota Roth fingerprint (KRFP) (Hua et al. 2022). Despite the existence of useful in silico models to identify neurotoxicants, a negative prediction alone is insufficient to draw a negative conclusion on neurotoxicity (Crofton et al. 2022). Gadaleta et al. applied AOPs in developing a QSAR tool, modeling the molecular initiating events (MIEs) in the existing AOP network for DNT. The prediction performance was compared to other reference QSAR standards, such as molecular descriptors and structural alerts, to return a comparable predictive performance (Gadaleta et al. 2022).

#### Physiologically based kinetic (PBK) models

In addition to QSAR models for addressing chemical and hazard characterization, significant effort and research focus on modeling key biological processes influencing ANT and DNT. Utilizing publicly available data, QSAR models for BBB and placental barrier have been developed employing methodologies such as Support Vector Machine (SVM), Multiple Linear Regression (MLR), and Artificial Neural Network (ANN). However, not all the BBB models developed adhere to the OECD QSAR model criteria (Masjosthusmann et al. 2018). Molecular descriptors like lipophilicity, polar surface area, and hydrogen bonding were instrumental in model development (Wang et al. 2011).

BBB permeability is a key factor in central nervous system exposure and its prediction is crucial in neurotoxicity research, given that a functional BBB prevents over 98% of chemicals from penetration (Goodwin and Clark 2005). Moreover, in silico ADME predictions can capture interspecies and intraspecies differences in ANT and DNT assessment, replacing traditional uncertainty factors with more precise chemical-specific adjustment factors (Kasteel and Westerink 2021).

To enhance confidence in in silico predictions for ANT and DNT, it is imperative to assess uncertainties within each model. Cronin and colleagues developed a schema to evaluate structural alerts predicting toxicity, proposing 12 criteria for assessing the quality and usability of an alert for a specific purpose (Cronin et al. 2022). Applying a similar scheme to ANT and DNT alerts can enhance trust and acceptance of these models. Evaluating uncertainties within a specific method generates alerts that allow higher confidence levels in decision-making.

An illustrative example of an AOP-based DNT model, considering uncertainties through a combination of in vitro and in silico approaches, has been presented by Spînu and colleagues. They developed a Bayesian hierarchical model of a simplified AOP network for DNT incorporating three common KEs: a reduction of brain-derived neurotrophic factor, a decrease of synaptogenesis, and a decrease in neuronal network formation. The training dataset comprised 88 chemicals from different sectors, and the data for the 3 KEs were gathered using in silico*/*in vitro methods. With an accuracy of 76%, their model classified compounds into three categories low, medium, and high probability of DNT potential (Spînu et al., 2019).

### In vitro models

One of the recognized drawbacks of using the animal model to study both DNT and ANT is that regulatory animal tests are not designed to generate mechanistic understanding and, therefore, do not necessarily provide an understanding of the mechanisms underlying toxicity. The utility of in vitro cell models lies in the ability to study detailed cellular and molecular mechanisms. The brain is a complex organ, so the challenge for in vitro models to be used in the context of neurotoxicology is to find ways to reproduce the complexity in terms of, e.g., cell types and intercellular communication. Moreover, many relevant processes are involved in neurotoxic outcomes, and one way to mimic the complexity of the in vivo brain is to plan test strategies based on data collection from a battery of assays (Fritsche et al. 2017).

The first cell-based in vitro models described include primary cultures of neurons and glial cells from animal origin, mainly rodent embryos and pups, and immortalized or tumor-derived cell lines (Abdulla and Campbell 1993; Harry et al. 1998). The latter two are suitable for in vitro testing because of their ease of handling and ability to expand rapidly but differ from primary cultures in several ways: (i) due to indefinite divisions, they can express unique gene patterns not found in any cell type in vivo, (ii) they may not have typical neural cell attributes or functions. In particular, immortalized cell lines of neuronal origin often lack ion channel and/or membrane receptor expression and activity, do not form effective synapses, and show unusual combinations of the neurotransmitters they produce (Edwards et al. 2007; LePage et al. 2005). As for primary cells, since they are of animal origin, molecular epitopes, gene expression, and physiological functions may differ from humans (Leist and Hartung 2013); however, they form excellent synaptic networks and contain various cell types of interest. For these reasons, they have been the main model used for mechanistic studies since now.

A move forward in in vitro NAMs came with the advent of human embryonic stem cells (hESCs) and human-induced pluripotent stem cells (hiPSCs) (Takahashi and Yamanaka 2006). hESCs and hiPSCs shifted the paradigm: they can expand and differentiate into virtually any brain cell type including both neurons and glial cells, providing an unlimited supply of cells from human origin and overcoming the issues of interspecies differences that are posed when using animal-derived primary cells (McComish and Caldwell 2018). Moreover, hiPSCs are considered identical to hESCs in terms of proliferation, differentiation abilities, and morphology, but override ethical issues concerning the use of hESCs which are derived from fertilized human embryos (Mallon et al. 2014). Unfortunately, the differentiation protocols are demanding, expensive, lengthy (at least 3–5 weeks of differentiation), and result in heterogeneous neuronal populations.

#### Mono-culture techniques

Neuron mono-cultures allow easy analysis of neuron-specific data, such as axon/dendrite growth, synapse formation, and all those transcriptional features and mechanisms of action that can be attributed to neurons alone. Furthermore, in the context of neurotoxicity testing, the neurotoxic effect of compounds on a specific cell type can be analyzed (Hopkins et al. 2015). An example of conditionally immortalized cells that provide a basis for chemical neurotoxicity testing is Lund human mesencephalic (LUHMES) cells; this cell line, derived from healthy 8-week-old human embryonic mesencephalic tissue, differentiates rapidly and homogeneously into mature dopaminergic neurons (Delp et al. 2018a). LUHMES cells are one of the models that are part of the DNT IVB (see chapter 3.3.5, UKN4 Assay—NeuriTox). This in vitro test method assesses one endpoint, the impairment in neurite outgrowth, after exposure to toxicants to evaluate both DNT, in terms of disturbances in the development of the nervous system and brain structures, and ANT in terms of direct damage to the adult nervous system. The advantage of the use of such a model is the possibility of high-throughput testing on a larger scale (Delp et al. 2018b). In contrast, this is a specific model for dopaminergic neurons, which represent < 1% of all neurons in the brain, so it cannot be considered representative of other neuronal types.

When it comes to hiPSCs-derived neurons, many protocols were developed to differentiate hiPSCs toward specific neuronal types and subtypes, reflecting features of a certain brain area (i.e., hippocampus; cortex) or with certain neurotransmitter characteristics (i.e., dopaminergic, glutamatergic, GABAergic). Logan and colleagues summarized the main protocols to generate neuronal and non-neuronal cells from hiPSCs reporting the most common strategies found in the literature (Logan et al. 2019).

Concerning glia, the species-specific differential gene expression profile and dissimilar expression of susceptibility genes for neurological disorders between animal models and human glial cells indicate the need for human-based models to better recapitulate the microenvironment of glial cells physiology and function (Gosselin et al. 2017). HiPSCs, hESCs, fibroblasts, peripheral blood mononuclear cells (PBMCs), and immortalized cell lines have been used to create mono-culture of human astrocytes and microglial cells in a laboratory setting, enabling the assessment of processes such as glutamate transport, inflammatory response, calcium responses, neurite outgrowth and maturation (Leventoux et al. 2020; Voulgaris et al. 2022; Speicher et al. 2019). Starting from hiPSCs, it is now possible to differentiate astrocytes, oligodendrocytes, microglia, and brain microvascular endothelial cells by adding a different sequence and combination of induction factors to the culture medium (Logan et al. 2019). These protocols are very recent, particularly those for the generation of microglia. Until 5 years ago, the only ways to obtain human microglia employed human monocytes cultured with astrocyte-conditioned medium (Leone et al. 2006) or PBMCs stimulated with a cocktail of four human recombinant cytokines (Etemad et al. 2012). Later, in 2017, a few papers were published that showed how to differentiate microglia from reprogrammed iPSCs to better mirror the developmental stages and ontogeny, considering that microglia derive from non-monocytic primitive myeloid cells (Muffat et al. 2016; Pandya et al. 2017; Haenseler et al. 2017; Douvaras et al. 2017). Despite possessing genes and functions unique to microglia, monolayers of in vitro microglial cell cultures can contain macrophages, lack regionality, and do not mirror the different subtypes found within the brain (Grabert et al. 2016). A non-exhaustive list of references reporting current protocols for human cell-derived in vitro models is provided in Table 3.

**Table 3:** A list of references reporting human cell-derived in vitro model systems from various sources

Neurons	Astrocytes	Oligodendrocytes	Microglia
hiPSc Glutamatergic Nehme et al. 2018 Boissart et al. 2013 GABAergic Yang et al. 2017 Dopaminergic Hartfield et al. 2014 Serotonergic Lu et al. 2016 Motor neurons Du et al. 2015	hiPSc Leventoux et al. 2020 Voulgaris et al. 2022	hiPSc Wang et al. 2013 Douvaras et al. 2014	hiPSc Speicher et al. 2019
	hESCs Byun et al. 2020	Immortalized cell lines De Kleijn et al. 2019	Primary macrophages Etemad et al. 2012
	Fibroblast-derived Meyer et al. 2014		Monocytes/PBMCs-derived Leone et al. 2006
	Immortalized cell lines Furihata et al. 2016		Immortalized cell lines Janabi et al. 1995
Immortalized cell lines Smirnova et al. 2016			

*hiPSc* human-induced pluripotent stem cells, *hESCs* human embryonic stem cells, *PBMCs* peripheral blood mononuclear cells.

#### Co-culture techniques

The main limitation of mono-culture models is the growth of a single cell type which cannot recapitulate specific physiological features due to insufficient cell–cell and cell–extracellular matrix interactions. Mono-cultures are, thus, far away from representing the human brain, but they can still be very useful in assessing DNT- and ANT-specific endpoints. The assessment of neurotoxicity cannot be discussed without considering the phenomenon of neuroinflammation. Several studies have shown that glial cells, especially astrocytes, are crucial for the formation of neuronal networks as pure neuronal cultures show limited bursting (Meneghello et al. 2015). This could be a problem when testing neurotoxicity outcomes, different MoAs identified as relevant to human neurotoxicity are related to neurotransmission (Masjosthusmann et al. 2018). The study by Tukker and colleagues showed that the addition of astrocytes in a glia/neuron ratio of 1:1, which is a near-physiological ratio, impacts the development of spontaneous neuronal network activity and bursting behavior (assessed by MEA) promoting neuronal network formation (Tukker et al. 2018). In addition to glial cells, the presence of both excitatory and inhibitory neurons also appears to play a role in the maturation of the network, with the optimal ratio defined as 1:5 inhibitory to excitatory neurons (Sahara et al. 2012). Moreover, aberrations in normal glial functions could affect neuron–glia communication possibly leading to toxicity and pathogenesis. At the same time, soluble molecules released in the medium and direct contact with other cell types are crucial for cultivating glia in their homeostatic state (Wenzel et al. 2023), this is a limitation observed when setting up mono-cultures of astrocytes, microglia oligodendrocytes. Mono-cultures of glial cells showed altered gene expression, morphology, and physical features (Dezonne et al. 2017; Bohlen et al. 2017).

#### Two-dimensional (2D) methods

The need to integrate the glial component into the neuronal cultures gave rise to multicellular systems. In 2D, cells can be co-cultured in the same environment, e.g., stimulating SH-SY5Y cells with BV2 culture supernatant (Guo et al. 2019), directly plating neurons on the glia monolayer (Shi et al. 2017), or simply plating primary cells derived from rodent embryos or pups without the addition of a cytostatic agent: this will allow glial cells to grow. Again, it is possible to plate cells on Petri dishes equipped with a porous membrane placed on a transwell; this method could somehow simulate the interaction between cells, but it is still limited in terms of direct cell–cell interaction. Transwell co-culture is particularly relevant for modeling the BBB (for an extensive review see Jackson et al. 2019). Finally, the glia–neuron sandwich co-culture could be a useful tool to assess interactions between different cell types based on the release of soluble factors secreted by both neurons and glia (Mancino et al. 2019). In terms of cost-effectiveness, simplicity, and the possibility to test neurotoxicity, these models are advantageous and, although some of them were developed with primary cells of animal origin, they could be set up also with hiPSCs-derived neurons and glial cells. Worth mentioning here also ex vivo brain slice cultures which are difficult to classify as 2D or 3D because, although they have a thickness, this is very different from the concept of 3D, which will be explored below. Slice cultures can preserve some elements of in vivo morphology, cytoarchitecture and anatomical connectivity; however, the process of generating slice cultures is morphologically damaging (for an extensive review see (Humpel 2015).

#### Three-dimensional (3D) methods

Multicellular systems could also be grown in 3D and we usually refer to them as spheroids or organoids. Spheroids are defined as 3D aggregates of multiple CNS cell types derived from neural progenitor cells (NPCs) cultured in non-adherent plates so that they cluster together and grow in suspension (Reynolds et al. 1992). An organoid is defined as “A 3D structure derived from pluripotent stem cells […] in which cells spontaneously self-organize into properly differentiated functional cell types and which recapitulates at least some functions of the organ” (Huch et al. 2017). Therefore, considering this definition, an organoid has three characteristics: (i) it is spatially organized in a way that resembles a human organ, not only at the cellular level, but also in terms of tissue structure and developmental trajectory (ii) it contains several organ-specific cell types, and (iii) it recapitulates a specific function. The main difference between spheroids and organoids is that the former typically lack distinctive cytoarchitecture (Hogberg and Smirnova 2022). The main pros and cons of 2D versus 3D cell culture methods are reported in Table 4.

**Table 4:** The pros and cons of 2D versus 3D cell culture methods

	2D	3D Spheroids	3D organoids		2D	3D spheroids	3D organoids
Easy handling/SOP				Cytoarchitecture			
Costs				High diversity of cell types			
Homogeneity				Control of environmental conditions			c
Reproducibility				Spatial organization			
Cell–cell interactions	a			Ease of manipulation for downstream analysis			
Cell–ECM interactions				High-throughput screening			d
Long-term culture		b		Brain Regionality			

**a** limited to side-by-side contact; **b** usually to a lesser extent when compared to 3D organoids; **c** typically cannot have both high complexity and high variable control of environmental conditions; **d** less amenable due to lack of standardization

*SOPs* standard operating procedure, *ECM* extracellular matrix

Lancaster and colleagues first allowed the differentiation of hiPSCs into organoids (Lancaster et al. 2013) and during the following years, this technique has been improved and refined. Given the rapid advances in the field and the continuous development of new experimental protocols, a recent article has attempted to clarify the nomenclature for nervous system organoids by emphasizing the self-organization feature of 3D cultures to derive unguided neural organoids, as opposed to regionalized neural organoids resembling regions or domains of the nervous system (Pașca et al. 2022). The combination of different regionalized brain organoids, called assembloids, is a further development of the three-dimensional in vitro culture technology that could allow the study of different aspects of the interactions between brain regions and domains soon (Marton and Pașca 2020).

As mentioned above, one of the key features of both spheroids and organoids is the diversity of cell types they contain. During differentiation, endoderm and mesoderm lineages are generally suppressed due to the patterning toward the ectodermal lineage. Therefore, microglia are generally assumed to be absent because of their non-neuroectodermal origin. Microglia originate in the yolk sac and reach the brain, where they mature, through vasculature (Nayak et al. 2014). This precludes the study of non-ectodermal cell types that play an important role in brain function and neurotoxic events, being microglia the resident immune cells of the brain. To overcome this limitation, it is possible to generate microglia from hiPSCs separately and then integrate them into brain organoids (Abud et al. 2017) or again to transplant microglia from primary origins (Popova et al. 2021). The benefits of integrating microglia into organoids have been multiple and have covered several domains: decrease of cellular stress, induction of transcriptional changes, facilitation of neural networks formation and maturation, also acting on bursting synchronization and frequency (Sabate-Soler et al. 2022).

Despite being very expensive, time-consuming, and requiring specialized knowledge, 3D organoids can accurately mimic physiological conditions, which are relevant for translational studies due to the human origin of the cells. 3D brain models are becoming increasingly complex to recapitulate human-relevant cellular processes and functionality. However, in neurotoxicology, the reproducibility of the system is a key point and it is, therefore, necessary to find a balance between complexity and simplicity to have robust, reproducible systems that can be used for high-throughput chemical screening. Spheroids are at a lower level of complexity than organoids, as shown in Table 4, but have the advantage of being more versatile as they can be used in low-/medium-throughput formats up to larger scale applications for screening purposes depending on manual pipetting or use of liquid handling systems. For these reasons, they are the most important model, though not the only one, part of DNT IVB, which will be discussed in more detail in the next chapter.

Some critical points represent a true challenge for the future—first of all, how to determine the in vitro age at which 3D brain spheroids and organoids correlate with the in vivo human adult brain. In vivo embryogenesis and organogenesis are processes that profoundly differ from in vitro spheroid and organoid formation (Bayir et al. 2019), since the in vitro environment, although highly uniform, could not match the real in vivo physiological conditions. Spheroids and neural organoids mostly mimic the early phases of embryonic development of the human brain (Trujillo et al. 2019; Porciúncula et al. 2021), thus are considered a suitable model for the study of DNT, but many questions are still open about a possible use in the context of ANT. This is one of the reasons why there are currently great differences between the development of methods for the study of DNT and ANT so there is a consistent methodological gap concerning the study of ANT. The “age issue” also applies to 2D cultures; tumor-derived cell lines can be isolated from young or old individuals, but this does not mean that they can be considered representative of the study of DNT or ANT, respectively. Furthermore, age-dependent phenotypes are present in vivo, but the length of time a cell line can be maintained in culture is limited. This is also a critical step for the study of chronic neurotoxicity using 2D cultures, as it is not possible to culture them for long periods. As with primary cultures, a maturation process can be identified. For example, primary rat hippocampal neurons are considered mature after at least 14 days in vitro (DIV), when the developed network is visible, morphological studies show the presence of mushroom-shaped synaptic spines, and functional studies show ion fluxes in response to pharmacological activation of receptors (Paoletti et al. 2013). However, this refers to the glutamatergic system and the timing may vary when other neuronal types or other brain regions are considered.

Finally, one issue regarding the use of hiPSCs in the study of ANT (both in 2D and 3D) concerns the process of de-differentiation through a stem cell-like stage. It has been demonstrated that after cell reprogramming, hiPSCs, and their derivatives are largely rejuvenated and have loose hallmarks of cellular aging typical of the original somatic cell source. These age-related cellular signatures include epigenetic features, energy metabolism, and other cellular mechanisms (Gladyshev 2016). Efforts have been made to develop alternative strategies that may be more suitable for ANT testing, such as direct reprogramming, in which cells are directly converted from one lineage to another without going through the pluripotent stage (Zhou-Yang et al. 2021; Mertens et al. 2018).

#### In vitro models for hazard and risk assessment: the example of DNT IVB

DNT represents an area where there is great interest in developing and applying NAMs for regulatory purposes. Thus, several international efforts have been made to address the need for a new framework that allows cost-effective and efficient screening and characterization of potential DNT hazards (Coecke et al. 2007; Lein et al. 2007) and to overcome the hurdles and questions that come alongside using a single in vitro test strategy such as: (i) elucidating interactions of several biological and toxicological mechanisms involved, (ii) causality from molecular interactions to neurodevelopmental disorders, and (iii) the extrapolation from obtained in vitro results to humans. The “Initial Recommendations on Evaluation of Data from the Developmental Neurotoxicity (DNT) In-Vitro Testing Battery”, released in the updated version in November 2023^12^ by the OECD, focuses on the use and interpretation of the DNT IVB and introduces a framework to enable regulatory use of the DNT IVB through an integrated approach to IATA, which represents a perfect tool to encompass and organize a variety of methods to address a specific case in a regulatory context (OECD 2020).

One NAM does not cover all key aspects of DNT. Thus, the establishment of the DNT IVB is based on the principle that the development of the nervous system in humans can be broken down into several key neurodevelopmental processes (KNDP) and that disruption of any of these KNDP may lead to DNT (Bal-Price et al. 2018). An overview of the updated DNT IVB is given in Table 5. It should be noted that many more NAMs related to DNT can be found in the literature and that this set of assays was selected based on three specific criteria: (i) complementarity, (ii) documentation (e.g., test description compatible with OECD guidance document 211 for describing non-guideline in vitro test methods (OECD 2017)) and (iii) readiness level (Patterson et al. 2021).

**Table 5:** Current status of the DNT IVB

Key event	Assay name	Cell type	Link to OECD appendix
Cell migration	UKN2 assay (cMINC)	hiPSC-derived NCCs	https://www.oecd.org/env/ehs/testing/appendix-b3-guidance-evaluation-of-data-developmental-neurotoxicity-in-vitro-testing-battery.pdf (accessed on Nov 2, 2023)
Neurite outgrowth (central)	UKN4 assay (NeuriTox)	LUHMES cells (differentiated into morphologically and biochemically mature dopamine-like neurons)	https://www.oecd.org/env/ehs/testing/appendix-b4-guidance-evaluation-of-data-developmental-neurotoxicity-in-vitro-testing-battery.pdf (accessed on Nov 2, 2023)
Neurite outgrowth (peripheral)	UKN5 assay (PeriTox)	hiPSC-derived iDRG	https://www.oecd.org/env/ehs/testing/appendix-b5-guidance-evaluation-of-data-developmental-neurotoxicity-in-vitro-testing-battery.pdf (accessed on Nov 2, 2023)
Precursor proliferation	Neurosphere Assay NPC1	hNPCs	https://www.oecd.org/chemicalsafety/testing/appendix-ba-guidance-evaluation-of-data-developmental-neurotoxicity-in-vitro-testing-battery.pdf (accessed on Nov 2, 2023)
Radial glia migration	Neurosphere assay NPC2a	hNPCs	https://www.oecd.org/chemicalsafety/testing/appendix-b2-guidance-evaluation-of-data-developmental-neurotoxicity-in-vitro-testing-battery.pdf (accessed on Nov 2, 2023)
Neuronal migration	Neurosphere assay NPC2b	hNPCs	
Oligodendrocyte migration	Neurosphere assay NPC2c	hNPCs	
Neuronal differentiation	Neurosphere assay NPC3	hNPCs	
Neurite outgrowth	Neurosphere assay NPC4	hNPCs	
Oligodendrocyte differentiation	Neurosphere assay NPC5	hNPCs	
Neurite outgrowth	Cortical initiation	Rat primary cortical neurons	https://www.oecd.org/chemicalsafety/testing/appendix-b6-guidance-evaluation-of-data-developmental-neurotoxicity-in-vitro-testing-battery.pdf (accessed on Nov 2, 2023)
Neurite maturation	Cortical maturation	Rat primary cortical neurons	https://www.oecd.org/chemicalsafety/testing/appendix-b7-guidance-evaluation-of-data-developmental-neurotoxicity-in-vitro-testing-battery.pdf (accessed on Nov 2, 2023)
Synaptogenesis	Cortical synapto	Rat primary cortical neurons	
Network formation assay (NFA)	Cortical MEA	Rat primary cortical neurons	https://www.oecd.org/chemicalsafety/testing/appendix-b8-guidance-evaluation-of-data-developmental-neurotoxicity-in-vitro-testing-battery.pdf (accessed on Nov 2, 2023)
Neurite outgrowth	hN initiation	hiPSC-derived neurons	https://www.oecd.org/chemicalsafety/testing/appendix-b9-guidance-evaluation-of-data-developmental-neurotoxicity-in-vitro-testing-battery.pdf (accessed on Nov 2, 2023)
Proliferation, cytotoxicity and apoptosis assay	hNP1 Apop	hNPCs	https://www.oecd.org/chemicalsafety/testing/appendix-b10-guidance-evaluation-of-data-developmental-neurotoxicity-in-vitro-testing-battery.pdf (accessed on Nov 2, 2023)
Proliferation, cytotoxicity and apoptosis assay	hNP1 Prolif	hNPCs	

*cMINC* circular migration inhibition of neural crest cells, *NCCs* neural crest cells, *hiPSC* human-induced pluripotent stem cell, *iDRG* immature dorsal root ganglia neurons, *hNPCs* human neural progenitor cells, *LUHMES* Lund human mesencephalic

The sensitivity of the predictions made by the current DNT IVB might be hampered by the lack of coverage of certain KNDP, such as stem cell differentiation toward neural progenitor cells, neural tube construction, and, importantly, the formation and function of neural networks (Blum et al. 2023). Together with toxicokinetic aspects, where for example a parent compound might not cause DNT, but a metabolite generated in vivo might be toxic, this could lead to false negative results. For these reasons, to date, a negative result after DNT IVB testing should not be interpreted as a lack of DNT potential and complementary in vitro assays should be added to the DNT IVB in the future to cover as many KNDP as possible and to solve uncertainties critical issues related to the in vitro methods used. Furthermore, there is a lack of empirical data correlating specific levels of alteration in the assays with known changes in in vivo neurodevelopmental outcomes. It should be noted that a classic OECD 34 validation of DNT IVB (i.e., interlaboratory testing of all DNT IVB tests on all compounds, including positive and negative molecules) has not been performed to date.

The DNT IVB has many potential regulatory applications, such as (i) identification or confirmation of possible DNT activity of compounds that were flagged by computational models, (ii) screening and prioritization of a large number of compounds for further testing, and (iii) specific testing of compounds with either inconclusive in vivo DNT data or novel data that causes concern (Sachana et al. 2021a). Furthermore, data obtained using NAMs in a weight-of-evidence-based approach have also already been used by the US EPA to waive the requirement of further guidelines in vivo testing (Dobreniecki et al. 2022), which is another important example of a possible application of the DNT IVB. The DNT IVB could provide data that are useful for hazard characterization and, by application of PBK modeling and IVIVE tools, benchmark doses obtained with the DNT IVB could be converted to administered equivalent doses that could then be used for risk assessment (Masjosthusmann et al. 2020; Blum et al. 2023). To increase confidence in the DNT IVB and to better clarify how it could be applied in a regulatory context, case studies in different regulatory settings should and are being performed with the DNT IVB in an AOP-informed IATA framework (Hernández-Jerez et al. 2021). Nevertheless, to increase predictive performance, the focus must be on cross-disciplinary approaches to further elucidate mechanisms underlying adverse effects on the nervous system. This should be combined with a regular revision of the DNT IVB as new assay techniques and further chemical test data become available (Sombers and Patisaul 2022; Crofton and Mundy 2021).

### *C. elegans* as a NAM model organism to study neurotoxicity

**Fig. 2:**
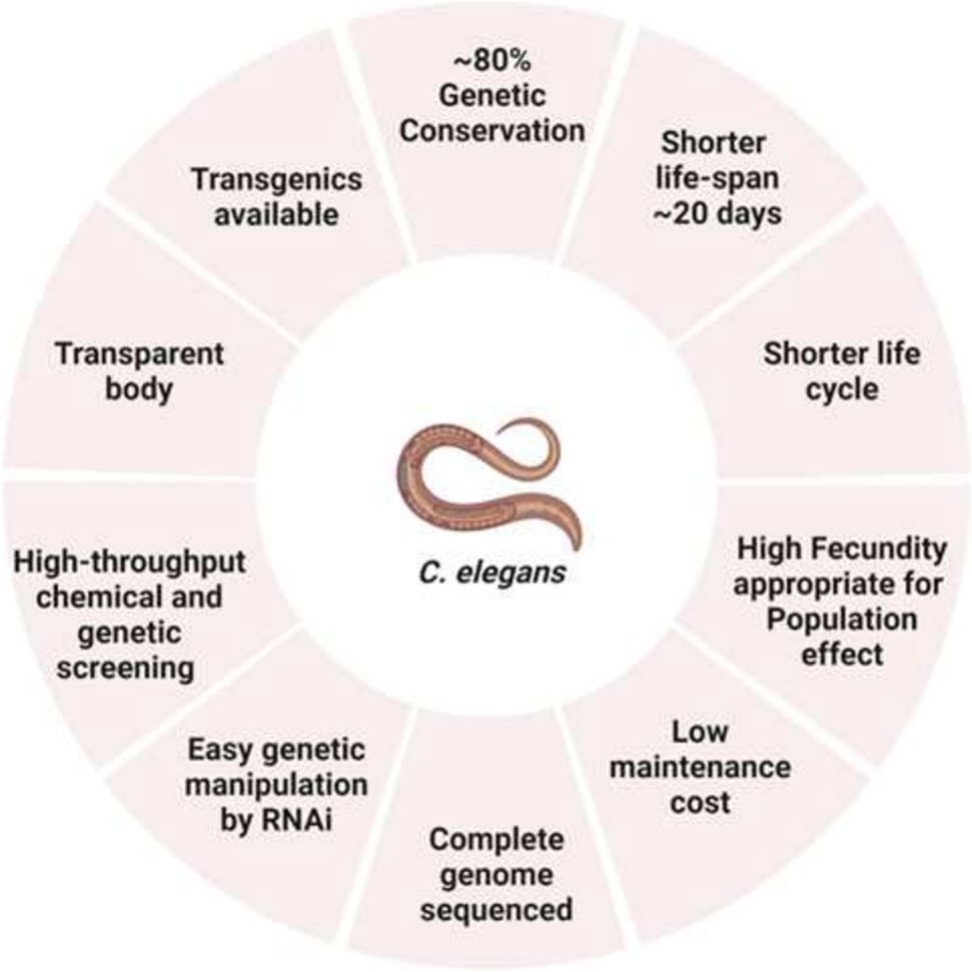
Advantages of the *C. elegans* model. Figure created with Biorender.com

For the regulatory assessment of DNT and ANT, in vivo mammalian models are considered the first choice as guidelines to date still rely primarily on mammalian animal testing, although the OECD, along with European and American regulatory agencies, are promoting the use of validated alternative test systems. Given the time and cost required to test an increasing number of compounds for DNT and ANT, the need for alternative models has arisen. The main challenge with NAMs is their reduced complexity compared to in vivo mammalian test systems, which limits their ability to answer open-ended questions. While NAMs are effective for screening and defining MoA, the complexity gap between in vivo and alternative systems has not yet been bridged. In addition to the possibility of creating batteries of many tests to assess different key processes for DNT and ANT, another strategy is the complementary use of animals that are not considered to be capable of suffering (partial replacement mentioned in Chapter 2). In this context, the *C. elegans* as NAM model is gaining popularity due to its simple genetics, conservation of key biological processes and genes (C. elegans Sequencing Consortium 1998), transparent body, short lifespan of 20 days, and cost-effective lab setup (Fig. 2). *C. elegans* is already a well-known experimental model with high sensitivity to diverse pollutants in soil and aquatic ecosystems, thus making it a great bridging model to assess environmental risk factors as per Environmental Risk Assessment (ERA) routines (Queirós et al. 2019).

Concerning neurotoxicity assessment, the entire neuron wire of *C. elegans* has been mapped: these organisms have 302 neurons, 56 glial cells, and 7600 synapses (White et al. 1986). The biochemical interactions of *C. elegans* neurons, synapses, and neurotransmitters are remarkably similar to those of mammals and well characterized (Bargmann 1998).

In addition, using genetic manipulation, transgenic animals expressing human genes with mutations for various diseases can be created to study the disease mechanism. High-throughput chemical screening, genetic screening, and behavioral assays can be easily achieved using this model system (Naranjo-Galindo et al. 2022). A recent study evaluated the morphological and behavioral endpoints of *C. elegans* in the context of reported AOP of neurodegenerative disorders and demonstrated the homology of human genes and associated proteins in the cholinergic and dopaminergic signaling system (Sammi et al. 2022). Also, an EFSA study conducted in 2018 showed that this model organism can be used as an alternative tool for ANT and MoA (Masjosthusmann et al. 2018).

About the endpoints addressed by the DNT IVB (reported in Table 5), implementing a similar battery of tests using *C. elegans* is yet to be developed. The study of neuronal cell proliferation from stem cells in *C. elegans* is well-established (Marchal and Tursun 2021). When reprogramming barriers like chromatin regulators are removed, germ cells can be reprogrammed to specific neuron subtypes (Kolundzic et al. 2018). Based on recent reports, exposing mercury to young *C. elegans* larvae displayed adult behavioral defects and DNT in dopaminergic neurons (Ruszkiewicz et al. 2018). Whereas lead neurotoxicity in young animals showed behavioral defects, cognitive defects, and neurodegeneration of cholinergic neurons upon aging (Ruszkiewicz et al. 2018). These studies can be extrapolated and compared to human outcomes, allowing us to develop *C. elegans* as a NAM for neurotoxicity assessment. The molecular mechanisms for neurite branching are similar to those found in mammals, with Netrin-1 and Anosmin-1 promoting the process in both nematodes and mammalian CNS neurons (Jin and Kim 2020). The cell fate of neuronal and glial stem cells has been fully mapped and there are 50 ectoderm-derived glia and 6 mesoderm-derived glia in the neuron–neuron junction, neuron–synapse junction, and sensory neuron junctions.

Epidemiological studies and meta-analyses suggest a relationship between pesticide exposure and neurodegenerative diseases such as PD and AD (EFSA 2014). Currently, *C. elegans* is widely used to study ANT caused by environmental toxicants like manganese, lead, arsenic, and mercury. Interestingly, high-throughput chemical genetic screening using *C. elegans* makes it possible to model PD, AD, and other neurodegenerative diseases. Like mammalian rodent models, *C. elegans* can be used to perform a variety of behavioral assays regulated by several classical neurotransmitters, which can further delineate the effect of neurotoxins (Iliff and Xu 2020). Neurodegeneration, protein aggregation, mitochondrial dysfunction, and reactive oxygen species formation are endpoints well explored in both PD and AD *C. elegans* models (Naranjo-Galindo et al. 2022; SenGupta et al. 2021). Worth mentioning, that steadily piling omics approaches in C. elegans AD and PD model allow for investigation of the molecular mechanism underlying DNT and ANT (SenGupta et al. 2022; Sánchez-Martínez et al. 2023).

However, the limiting factors associated with this model system are the absence of tissues, the BBB, and the circulatory system. In particular, while *C. elegans* has advantages for certain types of studies, it may not fully mimic the complexity of mammalian neurobiology due to phylogenetic distance.

### Zebrafish as a NAM model organism to study neurotoxicity

**Fig. 3:**
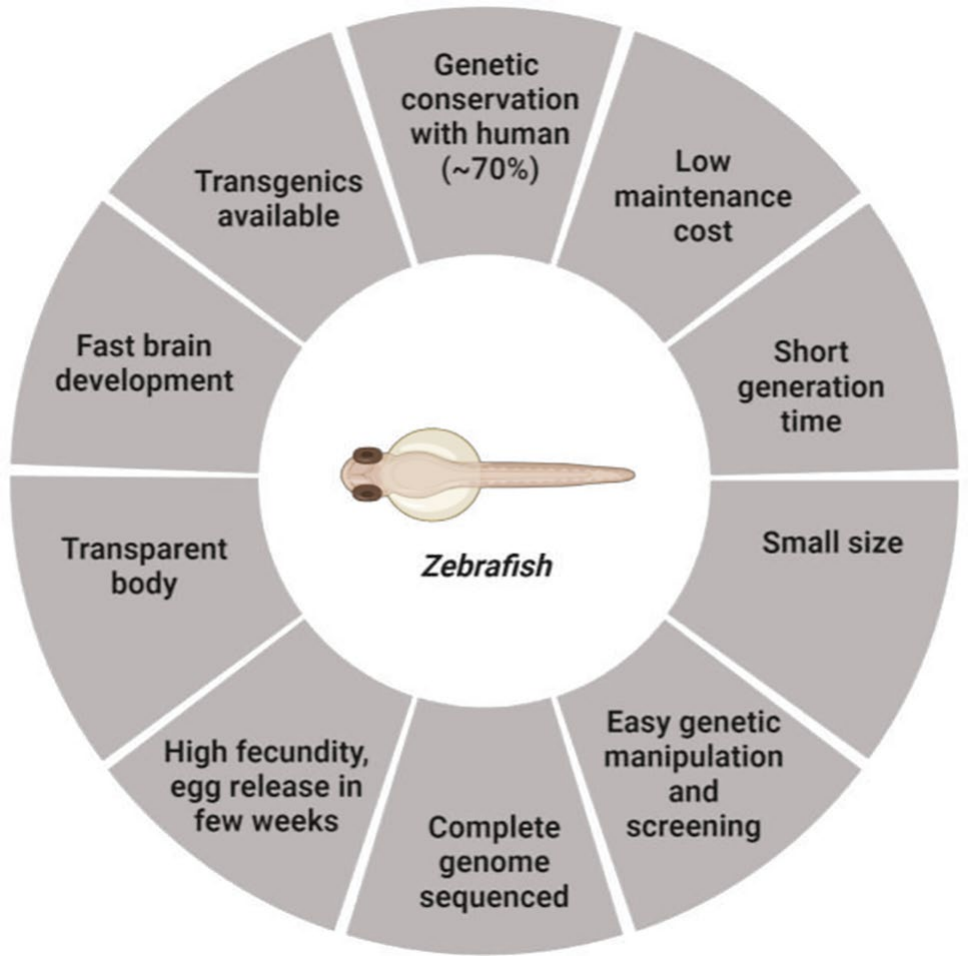
Advantages of the zebrafish model. Figure created with Biorender.com

Zebrafish (Danio rerio) is a small freshwater teleost fish that,over the years, has become an important model in biology, toxicology, human diseases, and physiology. Figure 3 summarizes the key features that make zebrafish an interesting organism for assessing DNT and ANT as an alternative to rodents (Parng et al. 2007; d’Amora and Giordani 2018).

The zebrafish genome was fully sequenced and shows approximately 70% homology with humans (de Esch et al. 2012). It has similar neuroendocrine hormones, neurotransmitters, and receptors involved in brain functions, such as learning and memory, (Takesono et al. 2022), although differences in expression patterns are observed when compared to human and rodents. Some areas of the zebrafish brain are similar to mammals and, despite the lack of a hippocampus, the lateral pallium appears to be functionally equivalent (Rodriguez et al. 2002).

The use of zebrafish to assess DNT is increasing because it avoids ethical constraints of rodent experiments. Zebrafish embryos and early larval stages, until free-swimming and independent feeding corresponding to 5-day post fertilization, could be considered as an alternative animal model^13^ (EFSA 2005). For ANT testing using adult fish, the legislation is more complex, butthe use of non-mammalian animals is still an important aspect of the 3Rs, as it falls within the definition of partial replacement.

Zebrafish motility and its response to various stimuli is an important behavioral indicator and could be used to understand neurobehavioral assessment in humans (Nishimura et al. 2015). Juvenile and adult zebrafish can mimic several human behaviors, which have been used to study both neurodevelopmental (e.g., autism, schizophrenia) and neurodegenerative (e.g., PD) disorders (for a list of zebrafish behavioral assays see Dasgupta et al. 2022). Unfortunately, despite the usefulness of behavioral assays for understanding toxicant effects, there is insufficient standardization of assay protocols or analysis methods between laboratories and it should also be recognized that there are clear anatomical differences between the mammalian and the zebrafish brain that need to be taken into account.

There are several examples of zebrafish models used to assess DNT and ANT. The microbiota–gut–brain axis is involved in a neurotoxicological contest and zebrafish is a powerful model (Bertotto et al. 2020) thanks to the high homology in the gastrointestinal tract (Goldsmith and Jobin 2012) and the ability to be directly exposed to environmental chemicals. Bisphenol A is an example of an environmental contaminant associated with adverse neurodevelopmental and endocrine effects, that has been studied using zebrafish (Catron et al. 2019a). These examples highlight the possibility of achieving a higher level of complexity compared to computational or in vitro methods, potentially assessing the intricate interplay with other organ systems (i.e., endocrine system and microbiota–gut–brain axis). The key to using zebrafish models is to determine which mechanisms are different from those in mammals and which are similar. Until these methods are adequately standardized and possibly even validated it is possible to use non-mammalian models in conjunction with classical mammalian models.

Perfluorooctane sulfonate, 17β-estradiol, ethanol, and acrylamide are just some of the other substances for which zebrafish has been used as a model to assess DNT, ANT, and behavioral toxicity (Tal et al. 2020; Catron et al. 2019b; Park et al. 2021; Fitzgerald et al. 2021). Neurotoxic effects in mammals of acrylamide (which induces apoptosis and demyelination) and ethanol (which affects neuronal proliferation, motor neuron survival, and optic nerve loss) were similarly assessed with the zebrafish model (Parng et al. 2007; Park et al. 2021). Another study shows how zebrafish estrogen pathways are homologous to those in rodents and humans. For this reason, zebrafish models have the potential for hazard assessment of endocrine disruptors and other developmentally neurotoxic chemicals (Takesono et al. 2022). Integrated multi-omics analysis using zebrafish has revealed the underlying molecular mechanism in response to polyfluoroalkyl substances (PFAS) used in the textile industry (Lee et al. 2021; Min et al. 2023). In conclusion, many qualitative endpoints for predicting DNT and ANT in mammals can be assessed in zebrafish, but direct comparison of zebrafish results to mammalian results still requires further validation.

## Conclusion

NAMs are defined as alternative approaches to classical animal testing; they include both non-animal approaches and test systems in phylogenetically lower species such as non-vertebrates and non-mammals. A PubMed search using the query “new approach methodology” returned 368 results from 2018 to the present.^14^ The growing importance of this argument to the scientific community is reflected in the number of scientific papers on the subject, despite its relative youth. For this reason, it would be desirable to standardize the definition of the term NAMs, so that it would be possible to define its use for further research.

Testing chemicals for their potential neurotoxic effects still comes alongside some hurdles:

- Minimal use of the non-obligatory regulatory in vivo test guideline studies with significant challenges in extrapolating findings from rodents to humans (Fritsche et al. 2017; Crofton and Mundy 2021; Tsuji and Crofton 2012);
- Time and cost-intensive nature of current in vivo and, to a lesser extent, alternative accepted methods (Fritsche et al. 2017; Crofton and Mundy 2021);
- Lack of data generation of one’s optimized alternative method causing low confidence in prediction and interpretation of data (Crofton et al. 2021);
- Limited funding left for developmental steps, leading to a wide range of alternative methods not necessarily being able to test many chemicals at once (Crofton et al. 2021; Coecke et al. 2007);
- Strict, but essential, guidelines on validation and regulation of alternative test methods that require a difficult process and high proof of concept, impending the encouragement of shifting to in vitro testing (Crofton and Mundy 2021; Grandjean and Landrigan 2006).

It is important to underline that the primary issue with animal testing is the relevance of data obtained to human health for regulatory purposes. Thus, the goal of new testing systems must be to address and improve this critical aspect, recognizing that mere replacement without achieving comparable scientific results will not be sufficient.

DNT and ANT assessment has to take into account a huge amount of variables: acute or chronic exposure, direct or indirect effects, reversibility or irreversibility of the effect, and the possibility of delayed effects (from hours or days to years). Of great relevance is the intricate interplay with other organ systems which makes it even more difficult to approach the complexity of the brain. Based on current knowledge, no single NAM can completely replace in vivo regulatory neurotoxicity tests alone because of its reduced complexity compared to in vivo mammalian systems. While NAMs are effective for screening and defining MoA, their ability to answer open-ended questions is limited.

A first attempt to bridge the complexity gap between in vivo and alternative systems is the creation of test batteries (e.g., the DNT IVB) that use different tests to address different endpoints that target key processes for neurotoxicity. Multiple assays can be combined into IATA. This requires an interdisciplinary approach based on the combined use of multiple sources of information so that different alternative methods can be used in concert to elucidate the mechanisms underlying adverse effects on the nervous system.

To date, several protocols have been published and are available to scientists; the hypotheses, specific aims, and study design determine the choice of one model over another. As far as in vitro methods, hiPSC-derived neurons are best suited to address different developmental stages of the nervous system to study DNT, rather than being representative of the adult nervous system, although progress is ongoing and recently protocols have been developed to generate fully mature, i.e., adult, neurons differentiated from hiPSC (Lin et al. 2023) and to perform direct reprogramming, to obtain adult neurons without going through the pluripotent stage (Mertens et al. 2018).

One complexity that many laboratories face is the reproducibility of the method. Bench experiments often have many variables that are difficult to control (e.g., variations in technicians, human error, machines, reagent lots), and reproducibility of data is also closely linked to the use of quality-controlled cells. Unfortunately, there is a lack of specific hands-on guidance on hiPSC quality control in the academic research environment (Li et al. 2015). Tigges and colleagues proposed a quality-controlled, two-step banking process to characterize the hiPSCs used in the laboratory with a panel of eight assays to ensure cell quality at moderate cost (Tigges et al. 2021); however, these tests are not routinely performed. As for the computational methods, they can theoretically be replicated exactly by an independent scientist, provided the raw data are available, the code is captured in a publicly available source, and the computational environment is dockerized; unfortunately, not all articles in the literature meet these requirements. This highlights the need to support data sharing, which is also critical for multi-omics approaches, by creating an infrastructure of tools, platforms, and software that can be accessed by researchers around the world.

As NAM models for neurotoxicity, *C. elegans* and zebrafish present comparable challenges. Despite their importance as tools for DNT and ANT assessment, there is an urgent need to harmonize and standardize methodologies by OECD Test Guidelines 426 and OECD Series on Testing and Assessment Number 377.^15^ As reported in a recent OECD case study entitled “The integrated methodologies for evaluating DNT to prioritize a class of flame retardants”^16^ zebrafish has already gained some popularity as a NAM for DNT, but its use could also be expanded to the ANT field (Dasgupta et al. 2022). *C. elegans* still has a long way to go as an accepted model, but a recent study by Sammi et al. effectively demonstrated a novel application of *C. elegans* under the AOP framework for neurotoxicity testing (Sammi et al. 2022) qualifying it as both a promising DNT and an ANT NAM model for neurotoxicity.

In conclusion, when working with NAMs, considering the number of available protocols covering a wide variety of endpoints and the questions that are still open, it is of critical importance to define the applicability domains to find a well-characterized system that suits the research question considering the accessibility of the material, throughput, and complexity. Finally, the keywords for the continuation of NAM studies should be standardization and reproducibility. The methods available in the literature are numerous, but these protocols must be standardized so that they can be used in different laboratories around the world obtaining strongly reproducible results. Standardization and reproducibility are also two central criteria for NAMs validation which is urgently needed. It is a long process, time- and cost-consuming, which needs a change of mindset first of all from researchers, but it is essential if we are to think about using NAMs for regulatory decision-making.

Standardization and reproducibility stand as pivotal criteria in the validation of NAMs, a process that is urgently needed. It is a long process, time- and cost-consuming, which necessitates a shift in mindset, particularly among researchers in academia. Financial support from stakeholders can play a crucial role in funding researchers to validate NAMs. Regulatory agencies in Europe already fund projects, including those focusing on interlaboratory transferability, a key point for results reproducibility. Progressing with standardization and reproducibility to achieve methods validation is essential if we are to think about using NAMs for regulatory decision-making.

## Data Availability

As this is a review, the authors do not consider the inclusion of a data availability statement to be applicable.

## Footnotes

1 https://eur-lex.europa.eu/legal-content/EN/TXT/?uri=CELEX:52013DC0859 (accessed on Jan 9, 2023).

2 https://op.europa.eu/en/publication-detail/-/publication/71ab691d-c9f6-11ed-a05c-01aa75ed71a1/language-en (accessed on Jan 9, 2023).

3 https://publications.parliament.uk/pa/cm200304/cmselect/cmsctech/172/17211.htm#n142 (accessed on Oct 6, 2023).

4 https://www.nal.usda.gov/animal-health-and-welfare/animal-use-alternatives (accessed on Sep 13, 2023).

5 https://www.ncbi.nlm.nih.gov/books/NBK4039/ (accessed on Oct 4, 2023).

6 https://jeodpp.jrc.ec.europa.eu/ftp/jrc-opendata/EURL-ECVAM/datasets/DBALM/LATEST/online/dbalm.html (accessed on Jan 19, 2024).

7 https://joint-research-centre.ec.europa.eu/eu-reference-laboratory-alternatives-animal-testing-eurl-ecvam/alternative-methods-toxicity-testing/validated-test-methods-health-effects_en (accessed on Jan 19, 2024).

8 https://one.oecd.org/document/ENV/JM/MONO(2020)21/en/pdf (accessed on Nov 3, 2023).

9 https://catalog.data.gov/dataset/alternatives-assessment-dashboard-hazard-database-version-1-0-generated-12-07-2018 (accessed on Jan 16, 2023).

10 https://ntp.niehs.nih.gov/data/index.html (accessed on Jan 16, 2023).

11 www.sapredictor.cn (accessed on Jan 16, 2023).

12 https://one.oecd.org/document/ENV/CBC/MONO(2023)13/en/pdf (accessed on Nov 3, 2023).

13 http://data.europa.eu/eli/dir/2010/63/2019-06-26 (accessed on Jun 1, 2023).

14 https://pubmed.ncbi.nlm.nih.gov/?term=%22new+approach+methodologies%22&sort=fauth&sort_order=asc&size=50 (accessed on Oct 31, 2023).

15 https://one.oecd.org/document/ENV/CBC/MONO(2023)13/en/pdf (accessed on Nov 3, 2023).

16 https://one.oecd.org/document/env/cbc/mono(2022)26/en/pdf (accessed on Nov 3, 2023).

## References

[CR1] Abdulla EM, Campbell IC (1993). In vitro tests of neurotoxicity. J Pharmacol Toxicol Methods.

[CR2] Abud EM, Ramirez RN, Martinez ES, Healy LM, Nguyen CHH, Newman SA, Yeromin AV, Scarfone VM, Marsh SE, Fimbres C, Caraway CA, Fote GM, Madany AM, Agrawal A, Kayed R, Gylys KH, Cahalan MD, Cummings BJ, Antel JP, Mortazavi A, Carson MJ, Poon WW, Blurton-Jones M (2017). iPSC-Derived Human Microglia-like Cells to Study Neurological Diseases. Neuron.

[CR3] Alimohammadi M, Meyburg B, Ückert AK, Holzer AK, Leist M (2023) EFSA Pilot Project on New Approach Methodologies (NAMs) for Tebufenpyrad Risk Assessment. Part 2. Hazard characterization and identification of the Reference Point. EFSA support Publ. EN-7794. 56 pp. 10.2903/sp.efsa.2023.EN-7794

[CR4] American Psychiatric Association (2013) Diagnostic and Statistical Manual of Mental Disorders, Fifth Edition. Arlington, VA, American Psychiatric Association

[CR5] Atkins JT, George GC, Hess K, Marcelo-Lewis KL, Yuan Y, Borthakur G, Khozin S, LoRusso P, Hong DS (2020). Pre-clinical animal models are poor predictors of human toxicities in phase 1 oncology clinical trials. Br J Cancer.

[CR6] Balls M (2002). Future improvements: replacement in vitro methods. ILAR J.

[CR7] Bal-Price A, Crofton KM, Sachana M, Shafer TJ, Behl M, Forsby A, Hargreaves A, Landesmann B, Lein PJ, Louisse J, Monnet-Tschudi F, Paini A, Rolaki A, Schrattenholz A, Suñol C, van Thriel C, Whelan M, Fritsche E (2015). Putative adverse outcome pathways relevant to neurotoxicity. Crit Rev Toxicol.

[CR8] Bal-Price A, Hogberg HT, Crofton KM, Daneshian M, FitzGerald RE, Fritsche E, Heinonen T, Hougaard Bennekou S, Klima S, Piersma AH, Sachana M, Shafer TJ, Terron A, Monnet-Tschudi F, Viviani B, Waldmann T, Westerink RHS, Wilks MF, Witters H, Zurich MG, Leist M (2018). Recommendation on test readiness criteria for new approach methods in toxicology: Exemplified for developmental neurotoxicity. Altex.

[CR9] Barbosa DJ, Capela JP, de Lourdes BM, Carvahlo F (2015). In vitro models for neurotoxicology research. Toxicol Res.

[CR10] Bargmann CI (1998). Neurobiology of the Caenorhabditis elegans genome. Science NY.

[CR11] Bayir E, Sendemir A, Missirlis YF (2019). Mechanobiology of cells and cell systems, such as organoids. Biophys Rev.

[CR12] Bell S, Abedini J, Ceger P, Chang X, Cook B, Karmaus AL, Lea I, Mansouri K, Phillips J, McAfee E, Rai R, Rooney J, Sprankle C, Tandon A, Allen D, Casey W, Kleinstreuer N (2020). An integrated chemical environment with tools for chemical safety testing. Toxicol in Vitro.

[CR13] Bertotto LB, Catron TR, Tal T (2020). Exploring interactions between xenobiotics, microbiota, and neurotoxicity in zebrafish. Neurotoxicology.

[CR14] Blum J, Masjosthusmann S, Bartmann K, Bendt F, Dolde X, Dönmez A, Förster N, Holzer AK, Hübenthal U, Keßel HE, Kilic S, Klose J, Pahl M, Stürzl LC, Mangas I, Terron A, Crofton KM, Scholze M, Mosig A, Leist M, Fritsche E (2023). Establishment of a human cell-based in vitro battery to assess developmental neurotoxicity hazard of chemicals. Chemosphere.

[CR15] Bohlen CJ, Bennett FC, Tucker AF, Collins HY, Mulinyawe SB, Barres BA (2017). Diverse Requirements for Microglial Survival, Specification, and Function Revealed by Defined-Medium Cultures. Neuron.

[CR16] Boissart C, Poulet A, Georges P, Darville H, Julita E, Delorme R, Bourgeron T, Peschanski M, Benchoua A (2013). Differentiation from human pluripotent stem cells of cortical neurons of the superficial layers amenable to psychiatric disease modeling and high-throughput drug screening. Transl Psychiatry.

[CR17] Byun JS, Lee CO, Oh M, Cha D, Kim WK, Oh KJ, Bae KH, Lee SC, Han BS (2020). Rapid differentiation of astrocytes from human embryonic stem cells. Neurosci Lett.

[CR18] C. elegans Sequencing Consortium (1998). Genome sequence of the nematode C elegans: a platform for investigating biology. Science NY.

[CR19] Capela JP, Carvalho FD (2022). A review on the mitochondrial toxicity of "ecstasy" (3,4-methylenedioxymethamphetamine, MDMA). Curr Res Toxicol.

[CR20] Catron TR, Keely SP, Brinkman NE, Zurlinden TJ, Wood CE, Wright JR, Phelps D, Wheaton E, Kvasnicka A, Gaballah S, Lamendella R, Tal T (2019). Host Developmental Toxicity of BPA and BPA Alternatives Is Inversely Related to Microbiota Disruption in Zebrafish. Toxicol Sci.

[CR21] Catron TR, Swank A, Wehmas LC, Phelps D, Keely SP, Brinkman NE, McCord J, Singh R, Sobus J, Wood CE, Strynar M, Wheaton E, Tal T (2019). Microbiota alter metabolism and mediate neurodevelopmental toxicity of 17β-estradiol. Sci Rep.

[CR22] Chushak YG, Shows HW, Gearhart JM, Pangburn HA (2018). In silico identification of protein targets for chemical neurotoxins using ToxCast in vitro data and read-across within the QSAR toolbox. Toxicol Res (camb).

[CR23] Coecke S, Goldberg AM, Allen S, Buzanska L, Calamandrei G, Crofton K, Hareng L, Hartung T, Knaut H, Honegger P, Jacobs M, Lein P, Li A, Mundy W, Owen D, Schneider S, Silbergeld E, Reum T, Trnovec T, Monnet-Tschudi F, Bal-Price A (2007). Workgroup report: incorporating in vitro alternative methods for developmental neurotoxicity into international hazard and risk assessment strategies. Environ Health Perspect.

[CR24] Costa LG (1998). Neurotoxicity testing: a discussion of in vitro alternatives. Environ Health Perspect.

[CR25] Costa LG, Giordano G (2007). Developmental neurotoxicity of polybrominated diphenyl ether (PBDE) flame retardants. Neurotoxicology.

[CR26] Crofton KM (2008). Thyroid disrupting chemicals: mechanisms and mixtures. Int J Androl.

[CR27] Crofton KM, Bassan A, Behl M, Chushak YG, Fritsche E, Gearhart JM, Marty MS, Mumtaz M, Pavan M, Ruiz P, Sachana M, Selvam R, Shafer TJ, Stavitskaya L, Szabo DT, Szabo ST, Tice RR, Wilson D, Woolley D, Myatt GJ (2022). Current status and future directions for a neurotoxicity hazard assessment framework that integrates in silico approaches. Comput Toxicol.

[CR28] Crofton KM and Mundy WR (2021) External Scientific Report on the Interpretation of Data from the Developmental Neurotoxicity In Vitro Testing Assays for Use in Integrated Approaches for Testing and Assessment. EFSA support Publ. EN-7794. 18, 6924E. 10.2903/SP.EFSA.2021.EN-6924

[CR29] Cronin MT (1996). Quantitative structure-Activity relationship (QSAR) analysis of the acute sublethal neurotoxicity of solvents. Toxicol in Vitro.

[CR30] Cronin MT, Bajot F, Enoch SJ, Madden JC, Roberts DW, Schwöbel J (2009). The in chemico-in silico interface: challenges for integrating experimental and computational chemistry to identify toxicity. Altern Lab Anim.

[CR31] Cronin MTD, Enoch SJ, Mellor CL, Przybylak KR, Richarz AN, Madden JC (2017). In Silico Prediction of Organ Level Toxicity: Linking Chemistry to Adverse Effects. Toxicol Res.

[CR32] Cronin MTD, Bauer FJ, Bonnell M, Campos B, Ebbrell DJ, Firman JW, Gutsell S, Hodges G, Patlewicz G, Sapounidou M, Spînu N, Thomas PC, Worth AP (2022). A scheme to evaluate structural alerts to predict toxicity: Assessing confidence by characterising uncertainties. Regul Toxicol Pharmacol.

[CR33] d'Amora M, Giordani S (2018). The Utility of Zebrafish as a Model for Screening Developmental Neurotoxicity. Front Neurosci.

[CR34] Dasgupta S, Simonich MT, Tanguay RL (2022). Zebrafish Behavioral Assays in Toxicology. Methods Mol Biol.

[CR35] de Esch C, Slieker R, Wolterbeek A, Woutersen R, de Groot D (2012). Zebrafish as potential model for developmental neurotoxicity testing: a mini review. Neurotoxicol Teratol.

[CR36] de Leeuw VC, van Oostrom CTM, Wackers PFK, Pennings JLA, Hodemaekers HM, Piersma AH, Hessel EVS (2022). Neuronal differentiation pathways and compound-induced developmental neurotoxicity in the human neural progenitor cell test (hNPT) revealed by RNA-seq. Chemosphere.

[CR37] De Kleijn KMA, Zuure WA, Peijnenborg J, Heuvelmans JM, Martens GJM (2019). Reappraisal of Human HOG and MO313 Cell Lines as a Model to Study Oligodendrocyte Functioning. Cells.

[CR38] Delp J, Gutbier S, Cerff M, Zasada C, Niedenführ S, Zhao L, Smirnova L, Hartung T, Borlinghaus H, Schreiber F, Bergemann J, Gätgens J, Beyss M, Azzouzi S, Waldmann T, Kempa S, Nöh K, Leist M (2018). Stage-specific metabolic features of differentiating neurons: Implications for toxicant sensitivity. Toxicol Appl Pharmacol.

[CR39] Delp J, Gutbier S, Klima S, Hoelting L, Pinto-Gil K, Hsieh JH, Aichem M, Klein K, Schreiber F, Tice RR, Pastor M, Behl M, Leist M (2018). A high-throughput approach to identify specific neurotoxicants/ developmental toxicants in human neuronal cell function assays. Altex.

[CR40] Delp J, Cediel-Ulloa A, Suciu I, Kranaster P, van Vugt-Lussenburg BM, Munic Kos V, van der Stel W, Carta G, Bennekou SH, Jennings P, Forsby A, Leist M (2021). Neurotoxicity and underlying cellular changes of 21 mitochondrial respiratory chain inhibitors. Arch Toxicol.

[CR41] Dezonne RS, Sartore RC, Nascimento JM, Saia-Cereda VM, Romão LF, Alves-Leon SV, de Souza JM, Martins-de-Souza D, Rehen SK, Gomes FC (2017). Derivation of Functional Human Astrocytes from Cerebral Organoids. Sci Rep.

[CR42] Dobreniecki S, Mendez E, Lowit A, Freudenrich TM, Wallace K, Carpenter A, Wetmore BA, Kreutz A, Korol-Bexell E, Friedman KP, Shafer TJ (2022). Integration of toxicodynamic and toxicokinetic new approach methods into a weight-of-evidence analysis for pesticide developmental neurotoxicity assessment: A case-study with DL- and L-glufosinate. Regul Toxicol Pharmacol.

[CR43] Douvaras P, Wang J, Zimmer M, Hanchuk S, O'Bara MA, Sadiq S, Sim FJ, Goldman J, Fossati V (2014). Efficient generation of myelinating oligodendrocytes from primary progressive multiple sclerosis patients by induced pluripotent stem cells. Stem Cell Rep.

[CR44] Douvaras P, Sun B, Wang M, Kruglikov I, Lallos G, Zimmer M, Terrenoire C, Zhang B, Gandy S, Schadt E, Freytes DO, Noggle S, Fossati V (2017). Directed Differentiation of Human Pluripotent Stem Cells to Microglia. Stem Cell Rep.

[CR45] Du ZW, Chen H, Liu H, Lu J, Qian K, Huang CL, Zhong X, Fan F, Zhang SC (2015). Generation and expansion of highly pure motor neuron progenitors from human pluripotent stem cells. Nat Commun.

[CR46] ECETOC (2014) TR 116 - Category approaches, read-across, (Q)SAR ECETOC Technical Reports 10.1016/B978-0-12-386454-3.00505-4.

[CR47] ECHA (2008). Guidance on information requirements and chemical safety assessment: QSARs and grouping of chemicals Guid Implement. Reach.

[CR48] Edwards MA, Loxley RA, Williams AJ, Connor M, Phillips JK (2007). Lack of functional expression of NMDA receptors in PC12 cells. Neurotoxicology.

[CR49] EFSA Panel on Animal Health and Welfare (2005). Opinion of the Scientific Panel on Animal Health and Welfare (AHAW) on a request from the Commission related to the aspects of the biology and welfare of animals used for experimental and other scientific purposes. EFSA J.

[CR50] EFSA Panel on Plant Protection Products and their Residues (2014). Scientific Opinion on the identification of pesticides to be included in cumulative assessment groups on the basis of their toxicological profile. EFSA J.

[CR51] El Yazal J, Rao SN, Mehl A, Slikker W (2001). Prediction of organophosphorus acetylcholinesterase inhibition using three-dimensional quantitative structure-activity relationship (3D-QSAR) methods. Toxicol Sci.

[CR52] Estrada E, Molina E, Uriarte E (2001). Quantitative structure-toxicity relationships using TOPS-MODE. 2. Neurotoxicity of a non-congeneric series of solvents. SAR QSAR Environ Res.

[CR53] Etemad S, Zamin RM, Ruitenberg MJ, Filgueira L (2012). A novel in vitro human microglia model: characterization of human monocyte-derived microglia. J Neurosci Methods.

[CR54] Fitzgerald JA, Könemann S, Krümpelmann L, Županič A, Vom Berg C (2021). Approaches to Test the Neurotoxicity of Environmental Contaminants in the Zebrafish Model: From Behavior to Molecular Mechanisms. Environ Toxicol Chem.

[CR55] Fritsche E, Crofton KM, Hernandez AF, Hougaard Bennekou S, Leist M, Bal-Price A, Reaves E, Wilks MF, Terron A, Solecki R, Sachana M, Gourmelon A (2017). OECD/EFSA workshop on developmental neurotoxicity (DNT): The use of non-animal test methods for regulatory purposes. Altex.

[CR56] Furihata T, Ito R, Kamiichi A, Saito K, Chiba K (2016). Establishment and characterization of a new conditionally immortalized human astrocyte cell line. J Neurochem.

[CR57] Gadaleta D, Spînu N, Roncaglioni A, Cronin MTD, Benfenati E (2022). Prediction of the Neurotoxic Potential of Chemicals Based on Modelling of Molecular Initiating Events Upstream of the Adverse Outcome Pathways of (Developmental) Neurotoxicity. Int J Mol Sci.

[CR58] Giordano G, Costa LG (2012). Developmental neurotoxicity: some old and new issues. ISRN Toxicol.

[CR59] Gladyshev VN (2016). Aging: progressive decline in fitness due to the rising deleteriome adjusted by genetic, environmental, and stochastic processes. Aging Cell.

[CR60] Goldsmith JR, Jobin C (2012). Think small: zebrafish as a model system of human pathology. J Biomed Biotechnol.

[CR61] Goodwin JT, Clark DE (2005). In silico predictions of blood-brain barrier penetration: considerations to "keep in mind". J Pharmacol Exp Ther.

[CR62] Gosselin D, Skola D, Coufal NG, Holtman IR, Schlachetzki JCM, Sajti E, Jaeger BN, O'Connor C, Fitzpatrick C, Pasillas MP, Pena M, Adair A, Gonda DD, Levy ML, Ransohoff RM, Gage FH, Glass CK (2017). An environment-dependent transcriptional network specifies human microglia identity. Science.

[CR63] Grabert K, Michoel T, Karavolos MH, Clohisey S, Baillie JK, Stevens MP, Freeman TC, Summers KM, McColl BW (2016). Microglial brain region-dependent diversity and selective regional sensitivities to aging. Nat Neurosci.

[CR64] Grandjean P, Landrigan PJ (2006). Developmental neurotoxicity of industrial chemicals. Lancet.

[CR65] Grigorev VYu, Raevskaya OE, Yarkov AV, Raevsky OA (2018). QSAR Modeling of Acute Neurotoxicity of Some Organic Solvents with Respect to Rodents. Biomed Chem Res Methods.

[CR66] Guo Y, Wei X, Yan H, Qin Y, Yan S, Liu J, Zhao Y, Jiang F, Lou H (2019). TREM2 deficiency aggravates α-synuclein-induced neurodegeneration and neuroinflammation in Parkinson's disease models. FASEB J.

[CR67] Haenseler W, Sansom SN, Buchrieser J, Newey SE, Moore CS, Nicholls FJ, Chintawar S, Schnell C, Antel JP, Allen ND, Cader MZ, Wade-Martins R, James WS, Cowley SA (2017). A Highly Efficient Human Pluripotent Stem Cell Microglia Model Displays a Neuronal-Co-culture-Specific Expression Profile and Inflammatory Response. Stem Cell Reports.

[CR68] Han Y, Zhang J, Hu CQ, Zhang X, Ma B, Zhang P (2019). In silico ADME and Toxicity Prediction of Ceftazidime and Its Impurities. Front Pharmacol.

[CR69] Harry GJ, Billingsley M, Bruinink A, Campbell IL, Classen W, Dorman DC, Galli C, Ray D, Smith RA, Tilson HA (1998). In vitro techniques for the assessment of neurotoxicity. Environ Health Perspect.

[CR70] Hartfield EM, Yamasaki-Mann M, Ribeiro Fernandes HJ, Vowles J, James WS, Cowley SA, Wade-Martins R (2014). Physiological characterisation of human iPS-derived dopaminergic neurons. PLoS ONE.

[CR71] Hartung T, McBride M (2011). Food for Thought on mapping the human toxome. Altex.

[CR72] Hartung T, FitzGerald RE, Jennings P, Mirams GR, Peitsch MC, Rostami-Hodjegan A, Shah I, Wilks MF, Sturla SJ (2017). Systems Toxicology: Real World Applications and Opportunities. Chem Res Toxicol.

[CR73] Helman G, Shah I, Williams AJ, Edwards J, Dunne J, Patlewicz G (2019). Generalized Read-Across (GenRA): A workflow implemented into the EPA CompTox Chemicals Dashboard. Altex.

[CR74] Hemmerich J, Ecker GF (2020). In silico toxicology: From structure-activity relationships towards deep learning and adverse outcome pathways. Wiley Interdiscip Rev Comput Mol Sci.

[CR75] Hendriksen CF (2009). Replacement, reduction and refinement alternatives to animal use in vaccine potency measurement. Expert Rev Vaccines.

[CR76] Hernández-Jerez A, Adriaanse P, Aldrich A, Berny P, Coja T, Duquesne S, Focks A, Marinovich M, Millet M, Pelkonen O, Pieper S, Tiktak A, Topping C, Widenfalk A, Wilks M, Wolterink G, Crofton K, Hougaard Bennekou S, Paparella M, Tzoulaki I (2021). Development of Integrated Approaches to Testing and Assessment (IATA) case studies on developmental neurotoxicity (DNT) risk assessment. EFSA J.

[CR77] Heyer DB, Meredith RM (2017). Environmental toxicology: Sensitive periods of development and neurodevelopmental disorders. Neurotoxicology.

[CR195] Hogberg HT, Smirnova L (2022). The future of 3D brain cultures in developmental neurotoxicity testing. Front Toxicol.

[CR78] Hopkins AM, DeSimone E, Chwalek K, Kaplan DL (2015). 3D in vitro modeling of the central nervous system. Prog Neurobiol.

[CR79] Hua Y, Cui X, Liu B, Shi Y, Guo H, Zhang R, Li X (2022). SApredictor: An Expert System for Screening Chemicals Against Structural Alerts. Front Chem.

[CR80] Huch M, Knoblich JA, Lutolf MP, Martinez-Arias A (2017). The hope and the hype of organoid research. Development.

[CR81] Humpel C (2015). Organotypic brain slice cultures: A review. Neuroscience.

[CR82] Iliff AJ, Xu XZS (2020). C. elegans: a sensible model for sensory biology. J Neurogenet.

[CR83] Jackson S, Meeks C, Vézina A, Robey RW, Tanner K, Gottesman MM (2019). Model systems for studying the blood-brain barrier: Applications and challenges. Biomaterials.

[CR84] Janabi N, Peudenier S, Héron B, Ng KH, Tardieu M (1995). Establishment of human microglial cell lines after transfection of primary cultures of embryonic microglial cells with the SV40 large T antigen. Neurosci Lett.

[CR85] Jiang C, Zhao P, Li W, Tang Y, Liu G (2020). In silico prediction of chemical neurotoxicity using machine learning. Toxicol Res.

[CR86] Jin H, Kim B (2020). Neurite Branching Regulated by Neuronal Cell Surface Molecules in Caenorhabditis elegans. Front Neuroanat.

[CR87] Kasteel EEJ, Westerink RHS (2021). Refining in vitro and in silico neurotoxicity approaches by accounting for interspecies and interindividual differences in toxicodynamics. Expert Opin Drug Metab Toxicol.

[CR88] Klose J, Pahl M, Bartmann K, Bendt F, Blum J, Dolde X, Förster N, Holzer AK, Hübenthal U, Keßel HE, Koch K, Masjosthusmann S, Schneider S, Stürzl LC, Woeste S, Rossi A, Covaci A, Behl M, Leist M, Tigges J, Fritsche E (2022). Neurodevelopmental toxicity assessment of flame retardants using a human DNT in vitro testing battery. Cell Biol Toxicol.

[CR89] Kolundzic E, Ofenbauer A, Bulut SI, Uyar B, Baytek G, Sommermeier A, Seelk S, He M, Hirsekorn A, Vucicevic D, Akalin A, Diecke S, Lacadie SA, Tursun B (2018). FACT Sets a Barrier for Cell Fate Reprogramming in Caenorhabditis elegans and Human Cells. Dev Cell.

[CR90] Kuhn M, Letunic I, Jensen LJ, Bork P (2016). The SIDER database of drugs and side effects. Nucleic Acids Res.

[CR91] Lancaster MA, Renner M, Martin CA, Wenzel D, Bicknell LS, Hurles ME, Homfray T, Penninger JM, Jackson AP, Knoblich JA (2013). Cerebral organoids model human brain development and microcephaly. Nature.

[CR92] Lee H, Sung EJ, Seo S, Min EK, Lee JY, Shim I, Kim P, Kim TY, Lee S, Kim KT (2021). Integrated multi-omics analysis reveals the underlying molecular mechanism for developmental neurotoxicity of perfluorooctanesulfonic acid in zebrafish. Environ Int.

[CR93] Lein P, Locke P, Goldberg A (2007). Meeting report: alternatives for developmental neurotoxicity testing. Environ Health Perspect.

[CR94] Leist M, Hartung T (2013). Inflammatory findings on species extrapolations: humans are definitely no 70-kg mice. Arch Toxicol.

[CR95] Leone C, Le Pavec G, Même W, Porcheray F, Samah B, Dormont D, Gras G (2006). Characterization of human monocyte-derived microglia-like cells. Glia.

[CR96] LePage KT, Dickey RW, Gerwick WH, Jester EL, Murray TF (2005). On the use of neuro-2a neuroblastoma cells versus intact neurons in primary culture for neurotoxicity studies. Crit Rev Neurobiol.

[CR97] Leventoux N, Morimoto S, Imaizumi K, Sato Y, Takahashi S, Mashima K, Ishikawa M, Sonn I, Kondo T, Watanabe H, Okano H (2020). Human Astrocytes Model Derived from Induced Pluripotent Stem Cells. Cells.

[CR98] Li F, Hu J, Xie K, He TC (2015). Authentication of experimental materials: A remedy for the reproducibility crisis?. Genes Dis.

[CR99] Li H, Zhao F, Cao F, Teng M, Yang Y, Qiu L (2019). Mitochondrial dysfunction-based cardiotoxicity and neurotoxicity induced by pyraclostrobin in zebrafish larvae. Environ Pollut.

[CR100] Lin W, Shiomoto S, Yamada S, Watanabe H, Kawashima Y, Eguchi Y, Muramatsu K, Sekino Y (2023). Dendritic spine formation and synapse maturation in transcription factor-induced human iPSC-derived neurons. iScience.

[CR101] Logan S, Arzua T, Canfield SG, Seminary ER, Sison SL, Ebert AD, Bai X (2019). Studying Human Neurological Disorders Using Induced Pluripotent Stem Cells: From 2D Monolayer to 3D Organoid and Blood Brain Barrier Models. Compr Physiol.

[CR102] Lu J, Zhong X, Liu H, Hao L, Huang CT, Sherafat MA, Jones J, Ayala M, Li L, Zhang SC (2016). Generation of serotonin neurons from human pluripotent stem cells. Nat Biotechnol.

[CR103] Makris SL, Raffaele K, Allen S, Bowers WJ, Hass U, Alleva E, Calamandrei G, Sheets L, Amcoff P, Delrue N, Crofton KM (2009). A retrospective performance assessment of the developmental neurotoxicity study in support of OECD test guideline 426. Environ Health Perspect.

[CR104] Mallon BS, Hamilton RS, Kozhich OA, Johnson KR, Fann YC, Rao MS, Robey PG (2014). Comparison of the molecular profiles of human embryonic and induced pluripotent stem cells of isogenic origin. Stem Cell Res.

[CR105] Malygin VV, Sokolov VB, Richardson RJ, Makhaeva GF (2003). Quantitative structure-activity relationships predict the delayed neurotoxicity potential of a series of O-alkyl-O-methylchloroformimino phenylphosphonates. J Toxicol Environ Health A.

[CR106] Mancino S, Serafini MM, Viviani B (2019) Neuron-Glia Interactions Studied with In Vitro Co-Cultures. In: Aschner M, Costa L (eds.) Cell Culture Techniques. Neuromethods, vol 145. Humana, New York, NY. 10.1007/978-1-4939-9228-7_5

[CR107] Marchal I, Tursun B (2021). Induced Neurons From Germ Cells in Caenorhabditis elegans. Front Neurosci.

[CR108] Marton RM, Pașca SP (2020). Organoid and Assembloid Technologies for Investigating Cellular Crosstalk in Human Brain Development and Disease. Trends Cell Biol.

[CR109] Marx-Stoelting P, Solano MLM, Aoyama H, Adams RH, Bal-Price A, Buschmann J, Chahoud I, Clark R, Fang T, Fujiwara M, Gelinsky M, Grote K, Horimoto M, Bennekou SH, Kellner R, Kuwagata M, Leist M, Lang A, Li W, Mantovani A, Makris SL, Paumgartten F, Perron M, Sachana M, Schmitt A, Schneider S, Schönfelder G, Schulze F, Shiota K, Solecki R (2021). 25th anniversary of the Berlin workshop on developmental toxicology: DevTox database update, challenges in risk assessment of developmental neurotoxicity and alternative methodologies in bone development and growth. Reprod Toxicol.

[CR110] Masjosthusmann S, Barenys M, El-Gamal M, Geerts L, Gerosa L, Gorreja A, Kühne B, Marchetti N, Tigges J, Viviani B, Witters H, Fritsche E (2018). Literature review and appraisal on alternative neurotoxicity testing methods. EFSA Support Publ.

[CR111] Masjosthusmann S, Blum J, Bartmann K, Dolde X, Holzer AK, Stürzl LC, Hagen Keßel E, Förster N, Dönmez A, Klose J, Pahl M, Waldmann T, Bendt F, Kisitu J, Suciu I, Hübenthal U, Mosig A, Leist M, Fritsche E (2020). Establishment of an a priori protocol for the implementation and interpretation of an in-vitro testing battery for the assessment of developmental neurotoxicity. EFSA Support Publ.

[CR112] Maurer LL, Philbert MA (2015). The mechanisms of neurotoxicity and the selective vulnerability of nervous system sites. Handb Clin Neurol.

[CR113] McComish SF, Caldwell MA (2018). Generation of defined neural populations from pluripotent stem cells. Philos Trans R Soc Lond B Biol Sci.

[CR114] Meneghello G, Verheyen A, Van Ingen M, Kuijlaars J, Tuefferd M, Van Den Wyngaert I, Nuydens R (2015). Evaluation of established human iPSC-derived neurons to model neurodegenerative diseases. Neuroscience.

[CR115] Mertens J, Reid D, Lau S, Kim Y, Gage FH (2018). Aging in a Dish: iPSC-Derived and Directly Induced Neurons for Studying Brain Aging and Age-Related Neurodegenerative Diseases. Annu Rev Genet.

[CR116] Meyer K, Ferraiuolo L, Miranda CJ, Likhite S, McElroy S, Renusch S, Ditsworth D, Lagier-Tourenne C, Smith RA, Ravits J, Burghes AH, Shaw PJ, Cleveland DW, Kolb SJ, Kaspar BK (2014). Direct conversion of patient fibroblasts demonstrates non-cell autonomous toxicity of astrocytes to motor neurons in familial and sporadic ALS. Proc Natl Acad Sci U S A.

[CR117] Mian P, Nolan B, van den Anker JN, van Calsteren K, Allegaert K, Lakhi N, Dallmann A (2021). Mechanistic Coupling of a Novel in silico Cotyledon Perfusion Model and a Physiologically Based Pharmacokinetic Model to Predict Fetal Acetaminophen Pharmacokinetics at Delivery. Front Pediatr.

[CR118] Min EK, Lee H, Sung EJ, Seo SW, Song M, Wang S, Kim SS, Bae MA, Kim TY, Lee S, Kim KT (2023). Integrative multi-omics reveals analogous developmental neurotoxicity mechanisms between perfluorobutanesulfonic acid and perfluorooctanesulfonic acid in zebrafish. J Hazard Mater.

[CR119] Muffat J, Li Y, Yuan B, Mitalipova M, Omer A, Corcoran S, Bakiasi G, Tsai LH, Aubourg P, Ransohoff RM, Jaenisch R (2016). Efficient derivation of microglia-like cells from human pluripotent stem cells. Nat Med.

[CR120] Naranjo-Galindo FJ, Ai R, Fang EF, Nilsen HL, SenGupta T (2022). C elegans as an Animal Model to Study the Intersection of DNA Repair Aging and Neurodegeneration. Front Aging.

[CR121] Nayak D, Roth TL, McGavern DB (2014). Microglia development and function. Annu Rev Immunol.

[CR122] Nehme R, Zuccaro E, Ghosh SD, Li C, Sherwood JL, Pietilainen O, Barrett LE, Limone F, Worringer KA, Kommineni S, Zang Y, Cacchiarelli D, Meissner A, Adolfsson R, Haggarty S, Madison J, Muller M, Arlotta P, Fu Z, Feng G, Eggan K (2018). Combining NGN2 Programming with Developmental Patterning Generates Human Excitatory Neurons with NMDAR-Mediated Synaptic Transmission. Cell Rep.

[CR123] Nelms MD, Mellor CL, Cronin MT, Madden JC, Enoch SJ (2015). Development of an in Silico Profiler for Mitochondrial Toxicity. Chem Res Toxicol.

[CR124] Nicolotti O, Benfenati E, Carotti A, Gadaleta D, Gissi A, Mangiatordi GF, Novellino E (2014). REACH and in silico methods: an attractive opportunity for medicinal chemists. Drug Discov Today.

[CR125] Nishimura Y, Murakami S, Ashikawa Y, Sasagawa S, Umemoto N, Shimada Y, Tanaka T (2015). Zebrafish as a systems toxicology model for developmental neurotoxicity testing. Congenit Anom.

[CR126] Ockleford C, Adriaanse P, Berny P, Brock T, Duquesne S, Grilli S, Hernandez-Jerez AF, Bennekou SH, Klein M, Kuhl T, Laskowski R, Machera K, Pelkonen O, Pieper S, Smith R, Stemmer M, Sundh I, Teodorovic I, Tiktak A, Topping CJ, Wolterink G, Angeli K, Fritsche E, Hernandez-Jerez AF, Leist M, Mantovani A, Menendez P, Pelkonen O, Price A, Viviani B, Chiusolo A, Ruffo F, Terron A, Bennekou SH (2017). Investigation into experimental toxicological properties of plant protection products having a potential link to Parkinson's disease and childhood leukaemia. EFSA J.

[CR127] OECD (1997) Test No. 424: Neurotoxicity Study in Rodents, OECD Guidelines for the Testing of Chemicals, section conclusion, OECD Publishing, Paris. 10.1787/9789264071025-en

[CR128] OECD (2007) Test No. 426: Developmental Neurotoxicity Study, OECD Guidelines for the Testing of Chemicals, section conclusion, OECD Publishing, Paris. 10.1787/9789264067394-en

[CR129] OECD (2014) Guidance on grouping of chemicals. OECD Series on Testing and Assessment, No. 194

[CR130] OECD (2017) Guidance Document for Describing Non-Guideline In Vitro Test Methods, OECD Series on Testing and Assessment, No. 211, OECD Publishing, Paris. 10.1787/9789264274730-en

[CR131] OECD (2018) Test No. 443: Extended One-Generation Reproductive Toxicity Study, OECD Guidelines for the Testing of Chemicals OECD Publishing, Paris. 10.1787/9789264185371-en

[CR132] OECD (2020) Overview of Concepts and Available Guidance related to Integrated Approaches to Testing and Assessment (IATA), OECD Series on Testing and Assessment, No. 329

[CR133] OECD (2021) Case study on the use of integrated approaches to testing and assessment for identification and characterisation of parkinsonian hazard liability of deguelin by an AOP-based testing and read across approach. OECD Series on Testing and Assessment, No. 326

[CR134] Pandya H, Shen MJ, Ichikawa DM, Sedlock AB, Choi Y, Johnson KR, Kim G, Brown MA, Elkahloun AG, Maric D, Sweeney CL, Gossa S, Malech HL, McGavern DB, Park JK (2017). Differentiation of human and murine induced pluripotent stem cells to microglia-like cells. Nat Neurosci.

[CR135] Paoletti P, Bellone C, Zhou Q (2013). NMDA receptor subunit diversity: impact on receptor properties, synaptic plasticity and disease. Nat Rev Neurosci.

[CR136] Park JS, Samanta P, Lee S, Lee J, Cho JW, Chun HS, Yoon S, Kim WK (2021). Developmental and Neurotoxicity of Acrylamide to Zebrafish. Int J Mol Sci.

[CR137] Parng C, Roy NM, Ton C, Lin Y, McGrath P (2007). Neurotoxicity assessment using zebrafish. J Pharmacol Toxicol Methods.

[CR138] Pașca SP, Arlotta P, Bateup HS, Camp JG, Cappello S, Gage FH, Knoblich JA, Kriegstein AR, Lancaster MA, Ming GL, Muotri AR, Park IH, Reiner O, Song H, Studer L, Temple S, Testa G, Treutlein B, Vaccarino FM (2022). A nomenclature consensus for nervous system organoids and assembloids. Nature.

[CR139] Patterson EA, Whelan MP, Worth AP (2021). The role of validation in establishing the scientific credibility of predictive toxicology approaches intended for regulatory application. Comput Toxicol.

[CR140] Pistollato F, Cavanaugh SE, Chandrasekera PC (2015). A human-based Integrated Framework For Alzheimer’s Disease Research. J Alzheimer's Dis.

[CR141] Pistollato F, de Gyves EM, Carpi D, Bopp SK, Nunes C, Worth A, Bal-Price A (2020). Assessment of developmental neurotoxicity induced by chemical mixtures using an adverse outcome pathway concept. Environ Health.

[CR142] Popova G, Soliman SS, Kim CN, Keefe MG, Hennick KM, Jain S, Li T, Tejera D, Shin D, Chhun BB, McGinnis CS, Speir M, Gartner ZJ, Mehta SB, Haeussler M, Hengen KB, Ransohoff RR, Piao X, Nowakowski TJ (2021). Human microglia states are conserved across experimental models and regulate neural stem cell responses in chimeric organoids. Cell Stem Cell.

[CR143] Porciúncula LO, Goto-Silva L, Ledur PF, Rehen SK (2021). The Age of Brain Organoids: Tailoring Cell Identity and Functionality for Normal Brain Development and Disease Modeling. Front Neurosci.

[CR144] Quaak I, Brouns MR, Van de Bor M (2013). The dynamics of autism spectrum disorders: how neurotoxic compounds and neurotransmitters interact. Int J Environ Res Public Health.

[CR145] Queirós L, Pereira JL, Gonçalves FJM, Pacheco M, Aschner M, Pereira P (2019). Caenorhabditis elegans as a tool for environmental risk assessment: emerging and promising applications for a "nobelized worm". Crit Rev Toxicol.

[CR146] Ransohoff RM (2018). All (animal) models (of neurodegeneration) are wrong. Are they also useful?. J Exp Med.

[CR147] Reynolds BA, Tetzlaff W, Weiss S (1992). A multipotent EGF-responsive striatal embryonic progenitor cell produces neurons and astrocytes. J Neurosci.

[CR148] Richard AM, Huang R, Waidyanatha S, Shinn P, Collins BJ, Thillainadarajah I, Grulke CM, Williams AJ, Lougee RR, Judson RS, Houck KA, Shobair M, Yang C, Rathman JF, Yasgar A, Fitzpatrick SC, Simeonov A, Thomas RS, Crofton KM, Paules RS, Bucher JR, Austin CP, Kavlock RJ, Tice RR (2021). The Tox21 10K Compound Library: Collaborative Chemistry Advancing Toxicology. Chem Res Toxicol.

[CR149] Roberts JR, Dawley EH, Reigart JR (2019). Children's low-level pesticide exposure and associations with autism and ADHD: a review. Pediatr Res.

[CR150] Rodríguez F, López JC, Vargas JP, Broglio C, Gómez Y, Salas C (2002). Spatial memory and hippocampal pallium through vertebrate evolution: insights from reptiles and teleost fish. Brain Res Bull.

[CR151] Russell WMS, Burch RL (1959). The Principles of Humane Experimental Technique. Med J Aust.

[CR152] Ruszkiewicz JA, Pinkas A, Miah MR, Weitz RL, Lawes MJA, Akinyemi AJ, Ijomone OM, Aschner M (2018). C. elegans as a model in developmental neurotoxicology. Toxicol Appl Pharmacol.

[CR153] Sabate-Soler S, Nickels SL, Saraiva C, Berger E, Dubonyte U, Barmpa K, Lan YJ, Kouno T, Jarazo J, Robertson G, Sharif J, Koseki H, Thome C, Shin JW, Cowley SA, Schwamborn JC (2022). Microglia integration into human midbrain organoids leads to increased neuronal maturation and functionality. Glia.

[CR154] Sachana M, Shafer TJ, Terron A (2021). Toward a Better Testing Paradigm for Developmental Neurotoxicity: OECD Efforts and Regulatory Considerations. Biology.

[CR155] Sachana M, Willett C, Pistollato F, Bal-Price A (2021). The potential of mechanistic information organised within the AOP framework to increase regulatory uptake of the developmental neurotoxicity (DNT) in vitro battery of assays. Reprod Toxicol.

[CR156] Sahara S, Yanagawa Y, O'Leary DD, Stevens CF (2012). The fraction of cortical GABAergic neurons is constant from near the start of cortical neurogenesis to adulthood. J Neurosci.

[CR157] Sammi SR, Jameson LE, Conrow KD, Leung MCK, Cannon JR (2022). Caenorhabditis elegans Neurotoxicity Testing: Novel Applications in the Adverse Outcome Pathway Framework. Front Toxicol.

[CR158] Sánchez-Martínez JD, Cifuentes A, Valdés A (2023). Omics approaches to investigate the neuroprotective capacity of a Citrus sinensis (sweet orange) extract in a Caenorhabditis elegans Alzheimer's model. Food Res Int.

[CR159] Schmidt CW (2014). NTP nonneoplastic lesion atlas: a new tool for toxicologic pathology. Environ Health Perspect.

[CR160] Schultz IR and Watanabe KH (2018) The Development of Quantitative AOPs. In: Garcia-Reyero N, Murphy CA (ed) A Systems Biology Approach to Adverse Outcome Pathways for Risk Assessment, 1st edn. Springer Cham. 10.1007/978-3-319-66084-4

[CR161] Schultz TW, Cronin MTD (2017). Lessons learned from read-across case studies for repeated-dose toxicity. Regul Toxicol Pharmacol RTP.

[CR162] SenGupta T, Palikaras K, Esbensen YQ, Konstantinidis G, Galindo FJN, Achanta K, Kassahun H, Stavgiannoudaki I, Bohr VA, Akbari M, Gaare J, Tzoulis C, Tavernarakis N, Nilsen H (2021). Base excision repair causes age-dependent accumulation of single-stranded DNA breaks that contribute to Parkinson disease pathology. Cell Rep.

[CR163] SenGupta T, Lefol Y, Lirussi L, Suaste V, Luders T, Gupta S, Aman Y, Sharma K, Fang EF, Nilsen H (2022). Krill oil protects dopaminergic neurons from age-related degeneration through temporal transcriptome rewiring and suppression of several hallmarks of aging. Aging.

[CR164] Shi Y, Yamada K, Liddelow SA, Smith ST, Zhao L, Luo W, Tsai RM, Spina S, Grinberg LT, Rojas JC, Gallardo G, Wang K, Roh J, Robinson G, Finn MB, Jiang H, Sullivan PM, Baufeld C, Wood MW, Sutphen C, McCue L, Xiong C, Del-Aguila JL, Morris JC, Cruchaga C, Fagan AM, Miller BL, Boxer AL, Seeley WW, Butovsky O, Barres BA, Paul SM, Holtzman DM (2017). ApoE4 markedly exacerbates tau-mediated neurodegeneration in a mouse model of tauopathy. Nature.

[CR165] Smirnova L, Hogberg HT, Leist M, Hartung T (2014). Developmental neurotoxicity - challenges in the 21st century and in vitro opportunities. Altex.

[CR166] Smirnova L, Harris G, Delp J, Valadares M, Pamies D, Hogberg HT, Waldmann T, Leist M, Hartung T (2016). A LUHMES 3D dopaminergic neuronal model for neurotoxicity testing allowing long-term exposure and cellular resilience analysis. Arch Toxicol.

[CR167] Soares MV, Mesadri J, Gonçalves DF, Cordeiro LM, Franzen da Silva A, Obetine Baptista FB, Wagner R, Dalla Corte CL, Soares FAA, Ávila DS (2022). Neurotoxicity induced by toluene: In silico and in vivo evidences of mitochondrial dysfunction and dopaminergic neurodegeneration. Environ Pollut.

[CR168] Sombers LA, Patisaul HB (2022). Virtual Issue: Neurotoxicology. ACS Chem Neurosci.

[CR169] Speicher AM, Wiendl H, Meuth SG, Pawlowski M (2019). Generating microglia from human pluripotent stem cells: novel in vitro models for the study of neurodegeneration. Mol Neurodegener.

[CR170] Spencer PS, Lein PJ (2024) Neurotoxicity. In: Encyclopedia of Toxicology, 4th edn. Academic Press, pp 727–740 10.1016/B978-0-12-824315-2.00548-0

[CR171] Spinu N, Bal-Price A, Cronin MTD, Enoch SJ, Madden JC, Worth AP (2019). Development and analysis of an adverse outcome pathway network for human neurotoxicity. Arch Toxicol.

[CR172] Spînu N, Cronin MTD, Lao J, Bal-Price A, Campia I, Enoch SJ, Madden JC, Mora Lagares L, Novič M, Pamies D, Scholz S, Villeneuve DL, Worth AP (2022). Probabilistic modelling of developmental neurotoxicity based on a simplified adverse outcome pathway network. Comput Toxicol.

[CR173] Takahashi K, Yamanaka S (2006). Induction of pluripotent stem cells from mouse embryonic and adult fibroblast cultures by defined factors. Cell.

[CR174] Takesono A, Kudoh T, Tyler CR (2022). Application of Transgenic Zebrafish Models for Studying the Effects of Estrogenic Endocrine Disrupting Chemicals on Embryonic Brain Development. Front Pharmacol.

[CR175] Tal T, Yaghoobi B, Lein PJ (2020). Translational Toxicology in Zebrafish. Curr Opin Toxicol.

[CR176] Tebby C, Gao W, Delp J, Carta G, van der Stel W, Leist M, Jennings P, Bois FY (2022). A quantitative AOP of mitochondrial toxicity based on data from three cell lines. Toxicol in Vitro.

[CR177] Tigges J, Bielec K, Brockerhoff G, Hildebrandt B, Hübenthal U, Kapr J, Koch K, Teichweyde N, Wieczorek D, Rossi A, Fritsche E (2021). Academic application of Good Cell Culture Practice for induced pluripotent stem cells. Altex.

[CR178] Trujillo CA, Gao R, Negraes PD, Gu J, Buchanan J, Preissl S, Wang A, Wu W, Haddad GG, Chaim IA, Domissy A, Vandenberghe M, Devor A, Yeo GW, Voytek B, Muotri AR (2019). Complex Oscillatory Waves Emerging from Cortical Organoids Model Early Human Brain Network Development. Cell Stem Cell.

[CR179] Tsuji R, Crofton KM (2012). Developmental neurotoxicity guideline study: issues with methodology, evaluation, and regulation. Congenit Anom.

[CR180] Tukker AM, Wijnolts FMJ, de Groot A, Westerink RHS (2018). Human iPSC-derived neuronal models for in vitro neurotoxicity assessment. Neurotoxicology.

[CR181] US EPA (1991) Guidelines for developmental toxicity risk assessment (EPA/600/FR-91/001). Fed Reg 56(234):63798–63826. https://www.epa.gov/risk/guidelines-developmental-toxicity-risk-assessment

[CR182] US EPA (1998) Health effects test guidelines: OPPTS 870.6200 neurotoxicity screening battery (EPA 712–C–98–238). https://nepis.epa.gov/Exe/ZyNET.exe/P100IRWB.TXT?ZyActionD=ZyDocument&Client=EPA&Index=1995+Thru+1999&Docs=&Query=&Time=&EndTime=&SearchMethod=1&TocRestrict=n&Toc=&TocEntry=&QField=&QFieldYear=&QFieldMonth=&QFieldDay=&IntQFieldOp=0&ExtQFieldOp=0&XmlQuery=&File=D%3A%5Czyfiles%5CIndex%20Data%5C95thru99%5CTxt%5C00000034%5CP100IRWB.txt&User=ANONYMOUS&Password=anonymous&SortMethod=h%7C-&MaximumDocuments=1&FuzzyDegree=0&ImageQuality=r75g8/r75g8/x150y150g16/i425&Display=hpfr&DefSeekPage=x&SearchBack=ZyActionL&Back=ZyActionS&BackDesc=Results%20page&MaximumPages=1&ZyEntry=1&SeekPage=x&ZyPURL#

[CR183] van der Stel W, Carta G, Eakins J, Darici S, Delp J, Forsby A, Bennekou SH, Gardner I, Leist M, Danen EHJ, Walker P, Jennings P (2020). Multiparametric assessment of mitochondrial respiratory inhibition in HepG2 and RPTEC/TERT1 cells using a panel of mitochondrial targeting agrochemicals. Arch Toxicol.

[CR184] Van der Stel W, Carta G, Eakins J, Delp J, Suciu I, Forsby A, Cediel-Ulloa A, Attoff K, Troger F, Kamp H, Gardner I, Zdrazil B, Moné MJ, Ecker GF, Pastor M, Gómez-Tamayo JC, White A, Danen EHJ, Leist M, Walker P, Jennings P, Hougaard Bennekou S (2021). New approach methods (NAMs) supporting read-across: Two neurotoxicity AOP-based IATA case studies. Altex.

[CR185] Voulgaris D, Nikolakopoulou P, Herland A (2022). Generation of Human iPSC-Derived Astrocytes with a mature star-shaped phenotype for CNS modeling. Stem Cell Rev Rep.

[CR186] Wang Z, Yan A, Li J (2011). In Silico Prediction of Blood Brain Barrier Permeability. SAR QSAR Environ Res.

[CR187] Wang S, Bates J, Li X, Schanz S, Chandler-Militello D, Levine C, Maherali N, Studer L, Hochedlinger K, Windrem M, Goldman SA (2013). Human iPSC-derived oligodendrocyte progenitor cells can myelinate and rescue a mouse model of congenital hypomyelination. Cell Stem Cell.

[CR188] Wenzel TJ, Le J, He J, Alcorn J, Mousseau DD (2023). Fundamental Neurochemistry Review: Incorporating a greater diversity of cell types, including microglia, in brain organoid cultures improves clinical translation. J Neurochem.

[CR189] White JG, Southgate E, Thomson JN, Brenner S (1986). The structure of the nervous system of the nematode Caenorhabditis elegans. Philos Trans R Soc Lond B Biol Sci.

[CR190] Wijeyesakere SJ, Wilson DM, Sue Marty M (2020). Prediction of cholinergic compounds by machine-learning. Comput Toxicol.

[CR191] Worth A, Lapenna F-G, S, Serafimova R, (2011). Applicability of QSAR analysis in the evaluation of developmental and neurotoxicity effects for the assessment of the toxicological relevance of metabolites and degradates of pesticide active substances for dietary risk assessment. EFSA Support Publ.

[CR192] Yang N, Chanda S, Marro S, Ng YH, Janas JA, Haag D, Ang CE, Tang Y, Flores Q, Mall M, Wapinski O, Li M, Ahlenius H, Rubenstein JL, Chang HY, Buylla AA, Südhof TC, Wernig M (2017). Generation of pure GABAergic neurons by transcription factor programming. Nat Methods.

[CR193] Zavala J, Freedman AN, Szilagyi JT, Jaspers I, Wambaugh JF, Higuchi M, Rager JE (2020). New Approach Methods to Evaluate Health Risks of Air Pollutants: Critical Design Considerations for In Vitro Exposure Testing. Int J Environ Res Public Health.

[CR194] Zhou-Yang L, Eichhorner S, Karbacher L, Böhnke L, Traxler L, Mertens J (2021). Direct Conversion of Human Fibroblasts to Induced Neurons. Methods Mol Biol.

